# Clustering of Activated CD8 T Cells Around Malaria-Infected Hepatocytes Is Rapid and Is Driven by Antigen-Specific Cells

**DOI:** 10.3389/fimmu.2019.02153

**Published:** 2019-09-20

**Authors:** Reka K. Kelemen, Harshana Rajakaruna, Ian A. Cockburn, Vitaly V. Ganusov

**Affiliations:** ^1^Institute of Science and Technology, Vienna, Austria; ^2^Genome Science and Technology Program, University of Tennessee, Knoxville, Knoxville, TN, United States; ^3^Department of Microbiology, University of Tennessee, Knoxville, Knoxville, TN, United States; ^4^Department of Immunology and Infectious Disease, John Curtin School of Medical Research, The Australian National University, Canberra, ACT, Australia

**Keywords:** CD8 T cell, Plasmodium, liver immunity, mathematical modeling, protection, vaccine

## Abstract

Malaria, a disease caused by parasites of the Plasmodium genus, begins when Plasmodium-infected mosquitoes inject malaria sporozoites while searching for blood. Sporozoites migrate from the skin via blood to the liver, infect hepatocytes, and form liver stages which in mice 48 h later escape into blood and cause clinical malaria. Vaccine-induced activated or memory CD8 T cells are capable of locating and eliminating all liver stages in 48 h, thus preventing the blood-stage disease. However, the rules of how CD8 T cells are able to locate all liver stages within a relatively short time period remains poorly understood. We recently reported formation of clusters consisting of variable numbers of activated CD8 T cells around *Plasmodium yoelii* (Py)-infected hepatocytes. Using a combination of experimental data and mathematical models we now provide additional insights into mechanisms of formation of these clusters. First, we show that a model in which cluster formation is driven exclusively by T-cell-extrinsic factors, such as variability in “attractiveness” of different liver stages, cannot explain distribution of cluster sizes in different experimental conditions. In contrast, the model in which cluster formation is driven by the positive feedback loop (i.e., larger clusters attract more CD8 T cells) can accurately explain the available data. Second, while both Py-specific CD8 T cells and T cells of irrelevant specificity (non-specific CD8 T cells) are attracted to the clusters, we found no evidence that non-specific CD8 T cells play a role in cluster formation. Third and finally, mathematical modeling suggested that formation of clusters occurs rapidly, within few hours after adoptive transfer of CD8 T cells, thus illustrating high efficiency of CD8 T cells in locating their targets in complex peripheral organs, such as the liver. Taken together, our analysis provides novel insights into and attempts to discriminate between alternative mechanisms driving the formation of clusters of antigen-specific CD8 T cells in the liver.

## 1. Introduction

Malaria is a life-threatening disease that is a result of red blood cell (erythrocyte) destruction by eukaryotic parasites of the *Plasmodium* genus. The majority of deaths (in recent years estimated to be about 500,000 annually) are among children, who have not yet developed immunity to the pathogen (1, 2). There are five species that infect humans: *P. falciparum, P. vivax, P. malariae, P. ovale*, and *P. knowlesi* (3). Three species of malaria parasites that are used as animal models for human malaria in mice are *P. yoelii, P. berghei*, and *P. chabaudi* (4). While there are similarities and differences in replication and pathogenesis of Plasmodium species in humans and mice, in this paper we focus solely on infection of mice with Plasmodium parasites.

The infection of the host is started by a mosquito, the vector between mammalian hosts, injecting the sporozoite form of parasites into the skin. Studies have estimated that the initial number of sporozoites entering the host is as low as 10–50 (5, 6), of which only a fraction succeed to migrate to the liver to start an infection of hepatocytes by forming liver stages (7–9). This liver stage of infection lasts for ~6.5 days in humans and about 2 days in mice (10–13). Because liver stage is asymptomatic, removal of all liver stages prevents clinical symptoms of malaria and thus is a highly desirable feature of an effective vaccine. Indeed, previous studies have shown that memory CD8 T cells are required for protection against a challenge with a relatively large number of sporozoites (14, 15) and that vaccination that induces exclusively memory CD8 T cells of a single specificity can mediate sterilizing protection against a sporozoite challenge (16–23). Antibodies and CD4 T cells may also contribute to protection in some circumstances, for example, following inoculation of sporozoites by mosquitoes in the skin (24, 25). Given that mouse liver contains about 1 − 2 × 10^8^ hepatocytes (26–28) and only a tiny proportion of these are infected the ability of memory CD8 T cells of a single specificity to locate and eliminate all liver stages within 48 h is remarkable. Yet, specific mechanisms by which CD8 T cells achieve such an efficiency remain poorly defined.

Recent studies utilizing fluorescently labeled sporozoites and activated Plasmodium-specific CD8 T cells and intravital microscopy revealed clustering of CD8 T cells near the parasite in the mouse livers whereby multiple T cells were located in close proximity (≤40 μm) of some liver stages (23, 29–31). Interestingly, we observed that clustering of CD8 T cells near the parasite results in a higher chances of parasite's death suggesting that clusters may increase the efficiency at which T cells eliminate the infection. Recent *in vivo* studies also found that the killing of virus-infected cells occurs faster when several CD8 T cells are near an infected cell (32) that is consistent with a previous report estimating that killing of targets *in vivo* follows the law of mass-action (33) (meaning that the rate of killing is directly proportional to the concentration of the killers and targets).

Clustering of CD8 T cells around Plasmodium liver stages in mice was not uniform as the majority of parasites had no T cells around them (at 6 h after T cell transfer), while some parasites were surrounded by 20–25 CD8 T cells (29). We have developed three alternative mathematical models aimed at explaining this observed variability in cluster formation and by fitting the models to a subset of the data concluded that the data are best explained by a model in which formation of clusters is driven by a positive feedback loop—clusters of a large size attract more CD8 T cells to the site of infection (29). Analysis of CD8 T cell movement in the liver suggested that there may be a bias toward the infection site (34). Additional experiments revealed a significant correlation between the rate at which new T cells locate the infection site and the number of CD8 T cells found in the cluster and independence of the per capita rate at which CD8 T cells leave the cluster from the cluster size—both observations were consistent with the “density-dependent” recruitment model.

Yet, our previous analysis did not investigate several other important issues of cluster formation. In particular, we did not fully determine the role of environmental variability in the formation of clusters around Plasmodium liver stages. Specifically, the observed correlation between entry rate into a cluster and cluster size could simply arise because some parasites may accidentally “attract” more T cells [e.g., due to higher induced inflammation or a higher blood flow rate (35, 36)]. In addition, we have observed that transfer of activated T cells, specific to Plasmodium, and T cells of irrelevant specificity resulted in co-clustering of T cells of two types (29); however, whether “non-specific” T cells contributed to the formation of clusters was not determined. Finally, our previous analyses did not determine the kinetics of the cluster formation by assuming that cluster size reaches a steady state by the time of imaging. In this paper with the use of mathematical modeling we provide additional insights into mechanisms by which clusters of activated CD8 T cells around Plasmodium liver stages are formed.

The main method of analysis used in this paper is comparison of several alternative mathematical models with experimental data. To ensure rigor of our analyses we provide a detailed description of experimental data and of the models in our Materials and Methods section. However, some readers may find it easier to directly read the Results section which provides a short overview of experiments (e.g., in figure legends) and references to the relevant mathematical models. Summary and implications of our results as well as limitations of our work are discussed in the Discussion section. Some additional results are also shown in Supplemental Information.

## 2. Materials and Methods

### 2.1. Data

**Figure 1:**
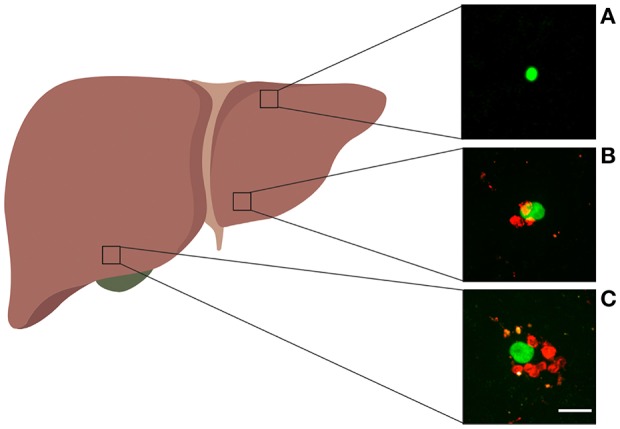
Examples of Plasmodium liver stages and clusters of activated CD8 T cells. Experiments were performed as shown in Figure S1A for control PyTCR cells, and examples of GFP-expressing malaria liver stages found in different parts of the liver are shown. **(A)** Liver stage with no CD8 T cells around, **(B)** liver stage with three CD8 T cell cells within 40 μm radius, **(C)** liver stage with 8 CD8 T cells within 40 μm radius. The white bar in panel C is 20 μm. Image of the liver was made with biorender.com.

In our analyses we used data from previously published work (29). Most data were generated using an experimental system with *in vivo* activated Py-specific TCR transgenic CD8 T cells (PyTCR) and in this paper we focus our analysis exclusively on CD8 T cells. Importantly, activation of CD8 T cells and expression of specific molecules (e.g., LFA1) is required for formation of liver-resident population (37, 38). For most of our analyses, data were from experiments involving infection of Balb/c mice with a high dose of Plasmodium yoelii (Py) sporozoites, expressing GFP; location of adoptively transferred activated CD8 T cells around GFP-expressing liver stages was then visualized using spinning disk confocal microscopy (29). Following our previous work we consider T cells located within 40 μm distance from the parasite as being close enough to recognize the infection; thus, all T cells within 40 μm from the parasite are called to form a “cluster” (Figure 1). How well the length of 40 μm represents the size of hepatocytes in mice remains unclear. 2D images of mouse hepatocytes suggested the diameter of 40–80 μm (39); however, measurements of the total volume of mouse hepatocytes of about $V_{h}=10^{4}\;\mu {\text{m}}^{3}$ (40) suggest a radius $r_{h}=\sqrt[{3}]{3V_{h}/\left(4\pi \right)}\approx 13\;\mu \text{m}$ for a “spherical” hepatocyte or cube edge length $r_{h}=\sqrt[{3}]{V_{h}}\approx 22\;\mu \text{m}$ for a “cubical” hepatocyte. A classical textbook on human liver anatomy cites the human hepatocyte volume of 10^4^ − 6 × 10^4^ μm^3^, corresponding to a cube edge of about 40 μm (35, p. 13). Despite these inconsistent estimates we consider T cells within 40 μm from the parasite to be close enough for recognition of the parasite.

Parasites in the imaging area were chosen at random and the number of CD8 T cells in the 40 μ*m* radius was calculated. Clustering of CD8 T cells around the parasite was measured in several alternative experiments. In one set of experiments, clustering of CD8 T cells around Py liver stages was performed by immunizing mice with radiation-attenuated Py sporozoites (RAS). In another set of experiments, clustering of T cells was observed following transfer of activated T cell receptor (TCR) transgenic CD8 T cells, specific to epitope located in Py circumsporozoite (CS) protein (CS_280-288_: SYVPSAEQI) denoted as PyTCR cells, or of TCR transgenic CD8 T cells, specific to an epitope in chicken ovalbumin (OVA_257–264_: SIINFEKL) denoted as OT1. Following infection with Py, PyTCR recognize the infection while OT1 cells serve as a control (non-specific to Py) T cells. PyTCR and OT1 cells were activated in similar *in vivo* experiments (using Vaccinia virus expressing CS or OVA epitopes) (29). Experiments involving co-transfer of PyTCR and OT1 cells were done in CB6 mice (F1 cross of Balb/c and C57BL/6 mice) (29).

In summary, the following datasets were used in the analysis:

1. Dataset #1. fluorescently labeled PyTCR cells (“PyTCR alone”) and PyTCR cells pre-treated with pertussis toxin ('PyTCR+PT') and transferred into mice infected with Py. This dataset was published before (29) but was not analyzed with mathematical models.
2. Dataset #2: naive or RAS-immunized mice that were infected with Py-GFP and clustering around the parasite was imaged using anti-CD8 antibody. This dataset was published before (29) and was only analyzed with models based on T cell-intrinsic clustering mechanisms (see below).
3. Dataset #3: fluorescently labeled PyTCR and OT1 cells transferred alone into individual mice (“PyTCR alone” and “OT1 alone”) or as 1:1 mixture (“PyTCR mix” and “OT1 mix”) to Py-infected mice. This dataset was published before (29) but was analyzed assuming that clustering of PyTCR and OT1 T cells was independent.
4. Dataset #4: fluorescently labeled PyTCR cells transferred into Py-infected mice and imaged at two different time points after T cell transfer. This dataset was generated for a previous study (29) but was not analyzed before with the use of mathematical models.

All datasets are made available as an online Supplemental Information to this paper to facilitate further independent analyses.

### 2.2. Mathematical Models

#### 2.2.1. Basic Mathematical Model for Clustering of One Cell Type

Previously we proposed a standard “birth-death" model to describe formation of clusters around Plasmodium liver stages (29). To be comprehensive, we describe this modeling framework here but then extend it to consider environmental variability in cluster sizes, co-clustering of CD8 T cells with different antigenic specificities, and kinetics of cluster formation. This modeling framework assumes that infection of hepatocytes by Plasmodium sporozoites occurs independently, i.e., there is no interactions between sporozoites infecting different hepatocytes. This assumption of independence is likely to be justified given that in our experiments (i) in general ~10^5^ sporozoites are injected i.v. into mice, (ii) only a fraction of these is expected to reach the liver (7–9, 41, 42), and (iii) mouse liver contains 1−2 × 10^8^ hepatocytes (26–28). Because in general in our experiments the number of Plasmodium-specific T cells exceeds the number of liver stages by 10- to 30-fold, it is likely that clustering of T cells around one parasite does not interfere or compete with T cells clustering around another parasite. In the model describing formation of clusters around Plasmodium liver stages by T cells of a single specificity we denote *P*_*k*_(*t*) as the probability to observe *k* T cells around the parasite at time *t* with *k* = 0, 1, 2, …*k*_max_. Increase in cluster size occurs at the “birth” (entry or cell division) rate λ_*k*_ (*k* = 0, 1, 2, …, *k*_max_) and decline in cluster size occurs due to “death” (or exit) rate μ_*k*_ (*k* = 1, 2, …, *k*_max_). The mathematical model describing the change in the probability *P*_*k*_(*t*) with time is given by the system of differential equations:

(1) $$\begin{array}{lll} \frac{\text{d}P_{0}\left(t\right)}{\text{d}t} & = & -\lambda_{0}P_{0}\left(t\right)+\mu_{1}P_{1}\left(t\right), \end{array}$$

(2) $$\begin{array}{lll} \frac{\text{d}P_{k}\left(t\right)}{\text{d}t} & = & -\left(\lambda_{k}+\mu_{k}\right)P_{k}\left(t\right)+\mu_{k+1}P_{k+1}\left(t\right)+\lambda_{k-1}P_{k-1}\left(t\right),\;k\geq 1. \end{array}$$

**Figure 2:**
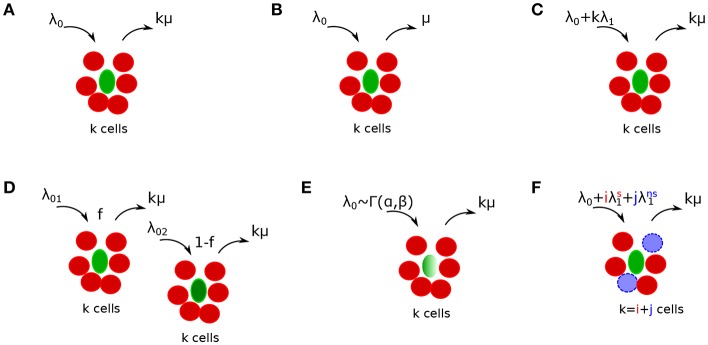
Schematics of alternative mathematical models of T cell cluster formation around *Plasmodium yoelii* (Py)-infected hepatocytes in mice. Py-specific T cells are labeled by red (disks), T cells of irrelevant specificity are colored by blue (dashed disks), and parasites are labeled by green (ovals). In the models the rate of T cell entry into a cluster is denoted as λ_*k*_ and rate of exit from the cluster is denoted as μ_*k*_. Mathematical models include a random entry/exit (Poisson) model (**A**, Equation (4), λ_*k*_ = λ_0_ and μ_*k*_ = *kμ*), a density-independent (DI) exit model (**B**, Equation (5), λ_*k*_ = λ_0_ and μ_*k*_ = μ), a density-dependent (DD) recruitment model (**C**, Equation (6), λ_*k*_ = λ_0_ + *kλ*_1_ and μ_*k*_ = *kμ*), a “two populations” model in which infected hepatocytes have either of two different “attractiveness” levels determined by λ_01_ and λ_02_ (**D**, Equation (11), μ_*k*_ = *kμ*), a “gamma” model, in which the entry rate into clusters is distributed according to a gamma distribution with α and β being the rate and shape parameters [**E**, Equations (12–15), μ_*k*_ = *kμ*], and finally a “co-clustering” model, in which clusters are formed by Plasmodium-specific T cells or T cells or irrelevant specificity (non-specific T cells) [**F**, Equations (12–15), $\lambda_{k}=\lambda_{0}+i\lambda_{1}^{s}+j\lambda_{1}^{ns}$ and μ_*k*_ = *kμ*]. For some of our analyses we characterized the model behavior using the ratio of entry to exit rates denoted as a relative entry rate θ_*k*_ = λ_*k*_/μ_*k*_.

By assuming different specific forms for the T cell entry (λ_*k*_) and exit (μ_*k*_) rates (e.g., see Figure 2 and below) the model can be solved numerically and fitted to the data using maximum likelihood method (see below). For some analyses we made a simplifying assumption that the distribution of cluster sizes reaches a steady state, and the steady state values for the probability to observe *k* CD8 T cells near a given liver stage $P_{k}^{*}$ is given by

(3) $$\begin{array}{l} P_{k}^{*}=P_{0}\frac{\prod_{i=0}^{k-1}\lambda_{i}}{\Pi_{i=1}^{k}\prod_{i}}, \end{array}$$

where $P_{0}^{*}$ is found by normalizing the total probability to one. By assuming steady state solutions it is in general impossible to estimate individual values for the rates of T cell entry into the cluster and exit from the cluster but we can estimate the ratio of the entry and exit rates, which we define as the relative entry rate θ_*k*_ = λ_*k*_/μ_*k*_. Validity of the steady state approximation is discussed in the Discussion section.

Mechanisms explaining the clustering of T cells around Plasmodium parasites in the liver can be broadly divided into two categories: T cell-intrinsic and T cell-extrinsic. In the T cell-intrinsic mechanism, the formation of clusters is driven exclusively by T cells and thus this mechanism ignores any potential differences in the variability in local liver environment. In the T cell-extrinsic mechanism, formation of clusters is driven exclusively due to variability in the liver environment near individual parasites, for example, due to a higher blood flow to some liver stages or a higher degree of inflammation that individual parasites may induce (35, 36). It is possible that ultimately both mechanisms may contribute to the cluster formation.

#### 2.2.2. Sub-models Assuming T Cell-Intrinsic Clustering Mechanisms

We consider several alternative models of how T cells may mediate formation of clusters around Plasmodium liver stages in mice (Figures 2A–C). Some of these models have been presented in our previous publication (29) and here are presented again for completeness. Our simplest random entry/exit (Poisson model) assumes that entry into the cluster and exit from the cluster occur randomly, i.e., λ_*k*_ = λ_0_ and μ_*k*_ = *kμ* where λ_0_ and μ are constants (Figure 2A). Solving Equation (3), the probability to observe *k* T cells around a parasite according to this random entry/exit model is then given by the Poisson distribution:

(4) $$\begin{array}{l} P_{k}^{*}=P_{0}^{*}\frac{\prod_{i=0}^{k-1}\lambda_{i}}{\prod_{i=1}^{k}\mu_{i}}=P_{0}^{*}\frac{\lambda_{0}^{k}}{\mu^{k}k!}=\frac{\theta_{0}^{k}}{k!}e^{-\theta_{0}}, \end{array}$$

where θ_0_ = λ_0_/μ.

We have shown previously that the Poisson model is often unable to describe distribution of cluster sizes of Plasmodium-specific CD8 T cells in the liver (29). One potential mechanism proposed to describe formation of large clusters is a “retention” model in which T cells which recognize the infection, are retained near the parasite. One version of such a model is a density-independent (DI) exit model (Figure 2B) in which the rate of T cell exit from a cluster declines with the number of T cells in the cluster, i.e., μ_*k*_ = *kμ*/*k* = μ for *k* > 0 and λ_*k*_ = λ_0_ for all *k*. Solving Equation (3), the probability to observe *k* T cells around a parasite according to the DI exit model is given by a geometric distribution:

(5) $$\begin{array}{l} P_{k}^{*}=P_{0}^{*}\frac{\prod_{i=0}^{k-1}\lambda_{i}}{\prod_{i=1}^{k}\mu_{i}}=P_{0}^{*}\frac{\lambda_{0}^{k}}{\mu^{k}}=\left(1-\theta_{0}\right)\theta_{0}^{k}, \end{array}$$

where θ_0_ = λ_0_/μ. There are other ways in which the total rate of T cell exit from the cluster μ_*k*_ could decline with cluster size *k* and in our additional analyses we tested two of such alternative models: a powerlaw model in which $\mu_{k}=k^{\alpha }\mu$ (defined for *k* > 0 with α and μ being constant) and an exponential model in which $\mu_{k}=k\mu e^{-\alpha k}$ (defined for *k* > 0 with α and μ being constant). When fitting these alternative retention models to experimental data we did not derive the steady state solutions but instead used numerical solutions of the basic mathematical model Equations (1) and (2).

An alternative mechanism for the formation of large clusters of CD8 T cells around the infection is an “attraction” model in which the rate of T cell entry into the cluster depends on cluster size. In this density-dependent (DD) recruitment model (Figure 2C) the entry rate into the cluster is given by λ_*k*_ = λ_0_ + λ_1_*k* while the total exit rate is density-dependent μ_*k*_ = *kμ*. This model also allows for the division of CD8 T cells in the cluster; therefore, the parameter λ_1_ denotes the combination of entry of new CD8 T cells to the cluster (dependent on cluster size) and division of T cells in the cluster. In our experiments with differentiated effector CD8 T cells we expect little cell division with several hours after T cell transfer. Solving Equation (3), the probability to observe *k* T cells around a parasite according to the DD recruitment model at the steady state is calculated numerically:

(6) $$\begin{array}{l} P_{k}^{*}=P_{0}^{*}\frac{\prod_{i=0}^{k-1}\lambda_{i}}{\prod_{i=1}^{k}\mu_{i}}=P_{0}^{*}\frac{\prod_{i=0}^{k-1}\lambda_{0}+i\lambda_{1}}{\mu^{k}k!}=P_{0}^{*}\frac{\prod_{i=0}^{k-1}\theta_{0}+i\theta_{1}}{k!}, \end{array}$$

where θ_0_ = λ_0_/μ and θ_1_ = λ_1_/μ and $P_{0}^{*}$ is found by normalizing Equation (6) assuming the maximal cluster size to be *k*_max_. In general, $\sum_{k=0}^{\infty }P_{k}^{*}\to \infty$ and therefore, the sum must be taken for a finite number of terms due to this reason (29).

To understand dynamics of cluster formation in the Poisson and DD recruitment models it is also useful and possible to derive the model describing the change in the average number of T cells around the parasite (average cluster size), $\langle k\rangle =\sum_{k=0}^{\infty }kP_{k}\left(t\right)$ using standard methods of physical chemistry (43)

(7) $$\begin{array}{l} \frac{\text{d}\langle k\rangle }{\text{d}t}=\lambda_{0}+\left(\lambda_{1}-\mu \right)\langle k\rangle , \end{array}$$

which is a standard birth-death process with immigration which for 〈*k*〉(0) = 0 has the solution

(8) $$\begin{array}{l} \langle k\rangle \left(t\right)=\frac{\lambda_{0}}{\lambda_{1}-\mu }\left({\text{e}}^{\left(\lambda_{1}-\mu \right)t}-1\right). \end{array}$$

In cases when λ_1_ > μ the average cluster size grows indefinitely with time. When λ_1_ < μ, which is often found in our analyses (see Results section), average cluster size at the steady state is given by

(9) $$\begin{array}{l} {\langle k\rangle }^{*}=\frac{\lambda_{0}}{\mu -\lambda_{1}}=\frac{\theta_{0}}{1-\theta_{1}}, \end{array}$$

where θ_0_ and θ_1_ are defined after Equation (6).

#### 2.2.3. Sub-models Assuming T Cell-Extrinsic Clustering Mechanisms (Environment)

An alternative mechanism for the formation of T cell clusters around Plasmodium-infected hepatocytes is proposed in this paper, namely, that the formation of clusters is driven by the ability of different parasites to “attract” T cells. For example, some parasites while traveling from the blood to hepatocyte or while replicating in the hepatocyte may induce higher degree of inflammation than other parasites, thus, potentially increasing the chance of finding such “inflamed” sites by T cells. Indeed, sporozoites are able to induce inflammation in the liver (36).

We consider two versions of the “environment” model in which T cell recruitment to sites is determined by the variability in parasite's “attractiveness.” In one such version, a two population model, we assume that there are parasites of two types found at frequencies *f* and 1 − *f*, and these parasites differ in the rate at which T cells find them (Figure 2D). The formation of clusters around parasites of a given parasite type is given by random entry/exit model with rates λ_01_ and λ_02_ while the rate of exit of T cells from the cluster is μ_*k*_ = *kμ*. Then assuming a steady state the probability to observe clusters of size *k* is given by

(10) $$\begin{array}{l} P_{k}^{*}=f\frac{\theta_{01}^{k}}{k!}e^{-\theta_{01}}+\left(1-f\right)\frac{\theta_{02}^{k}}{k!}e^{-\theta_{02}}, \end{array}$$

where θ_01_ = λ_01_/μ and θ_02_ = λ_02_/μ. Alternatively, the rate at which T cells find parasites could be given by a continuous function, and we tested a model in which entry rate into the cluster is given by a gamma distribution $g\left(\lambda_{0};\alpha ,\beta \right)=\frac{\beta^{\alpha }\lambda_{0}^{\alpha -1}e^{-\lambda_{0}\beta }}{\Gamma \left(\alpha \right)}$, i.e., the probability for T cells to have an entry rate in the interval (λ_0_, λ_0_ + dλ_0_) is *g*(λ_0_; α, β)dλ_0_. The probability to observe a cluster of size *k* given that clustering around a parasite “attracting” T cells at a rate λ_0_ follows a Poisson model is given by an integral

(11) $$\begin{array}{l} P_{k}^{*}=\int_{0}^{\infty }\frac{\lambda_{0}^{k}}{\mu^{k}k!}e^{-\lambda_{0}/\mu }\times \frac{\beta^{\alpha }\lambda_{0}^{\alpha -1}e^{-\lambda_{0}\beta }}{\Gamma \left(\alpha \right)}d\lambda_{0}\\ ={\left(\mu^{-1}+\beta \right)}^{-\left(\alpha +k\right)}\frac{\beta^{\alpha }\Gamma \left(\alpha +k\right)}{\mu^{k}k!\Gamma \left(\alpha \right)}, \end{array}$$

where α and β are the shape and rate parameters of the Gamma distribution, respectively, and Γ(α) = (α − 1)!.

#### 2.2.4. A Basic Mathematical Model for Clustering of Two Cell Types

In some of our experiments we tracked clustering of T cells of two specificities: one type of T cells was specific to Plasmodium sporozoites (PyTCR) and another type of T cells was specific to irrelevant antigen (OT1). To quantify the kinetics of clustering of Plasmodium-specific (PyTCR) and non-specific T cells (OT1) around Plasmodium-infected hepatocytes, we extended our basic model Equations (1), (2) to include two types of cells, *t*_1_ and *t*_2_, in the cluster to formulate a co-clustering model (Figure 2F). We define *P*_*ij*_(*t*) as the probability to observe *i* cells of type 1 and *j* cells of type 2 in a given cluster. Then the rate at which new T cells of type *x*, where *x* = *t*_1_, *t*_2_ enter the cluster with *i* T cells of type 1 and *j* T cells of type 2 is $\lambda_{ij}^{x}$. Similarly, $\mu_{ij}^{x}$ is the rate of exit of T cell of type *x* from a cluster with (*i, j*) T cells. The dynamics of the probability to observe a cluster with (*i, j*) T cells is given by equations

(12) $$\begin{array}{lll} \frac{\text{d}P_{00}\left(t\right)}{\text{d}t} & = & -\left(\lambda_{00}^{t_{1}}+\lambda_{00}^{t_{2}}\right)P_{00}\left(t\right)+\mu_{10}^{t_{1}}P_{10}\left(t\right)+\mu_{01}^{t_{2}}P_{01}\left(t\right), \end{array}$$

(13) $$\begin{array}{l} \frac{\text{d}P_{01}(t)}{\text{d}t}=-(\lambda_{01}^{t_{1}}+\lambda_{01}^{t_{2}}+\mu_{01}^{t_{2}})P_{01}(t)+\lambda_{00}^{t_{2}}P_{00}(t)+\mu_{11}^{t_{1}}P_{11}(t)\\ \;+\mu_{02}^{t_{2}}P_{02}(t), \end{array}$$

(14) $$\begin{array}{l} \frac{\text{d}P_{10}(t)}{\text{d}t}=-(\lambda_{00}^{t_{1}}+\lambda_{01}^{t_{2}}+\mu_{10}^{t_{1}})P_{10}(t)+\lambda_{00}^{t_{1}}P_{00}(t)+\mu_{11}^{t_{2}}P_{11}(t)\\ \;+\mu_{20}^{t_{1}}P_{20}(t), \end{array}$$

(15) $$\begin{array}{l} \frac{\text{d}P_{ij}(t)}{\text{d}t}=-(\lambda_{ij}^{t_{1}}+\lambda_{ij}^{t_{2}})P_{ij}(t)+\lambda_{(i-1)j}^{t_{1}}P_{(i-1)j}(t)+\lambda_{i(j-1)}^{t_{2}}P_{i(j-1)}(t)\\ \;+\mu_{(i+1)j}^{t_{1}}P_{(i+1)j}(t)+\mu_{i(j+1)}^{t_{2}}P_{i(j+1)}(t),i,j=2..k_{\max}. \end{array}$$

The dynamics of the probability *P*_*ij*_(*t*) can be simulated by assuming different functional forms for the entry and exit rates (Table 1). For example, when the entry rate into the cluster is independent of the cluster size or cell type, $\lambda_{ij}^{t_{1}}=\lambda_{ij}^{t_{2}}=\lambda_{0}=\text{const}$, and the exit rate is dependent on the number of T cells of a given specificity present near the parasite, $\mu_{ij}^{t_{1}}=i\mu$ and $\mu_{ij}^{t_{2}}=j\mu$ where μ = const, clustering of T cells is independent and is described at the steady state by the Poisson distribution (results not shown). Another model is when the entry rate of T cells into the cluster is dependent only on the number of specific T cells (*t*_1_) already in the cluster: $\lambda_{ij}^{t_{1}}=\lambda_{ij}^{t_{2}}=\lambda_{0}+i\lambda_{1}$ with exit rates being similar to the random entry/exit model described above.

**Table 1:** Defining alternative models for co-clustering of Plasmodium-specific (*s*) and non-specific (*ns*) T cells around Plasmodium liver stages.

**Model**	**$\lambda_{ij}^{s}$**	**$\lambda_{ij}^{ns}$**	**$\mu_{ij}^{s}$**	**$\mu_{ij}^{ns}$**
Random entry/exit	λ_0_	λ_0_	μ*i*	μ*j*
Equal recruitment	λ_0_ + λ_1_ · (*i* + *j*)	λ_0_ + λ_1_ · (*i* + *j*)	μ*i*	μ*j*
Only specific T cells recruit	λ_0_ + λ_1_ · *i*	λ_0_ + λ_1_ · *i*	μ*i*	μ*j*
Only non-specific T cells recruit	λ_0_ + λ_1_ · *j*	λ_0_ + λ_1_ · *j*	μ*i*	μ*j*
Basic entry rates are type-specific and only specific T cells recruit	$\lambda_{0}^{s}+\lambda_{1}\cdot i$	$\lambda_{0}^{ns}+\lambda_{1}\cdot i$	μ*i*	μ*j*
Both T cell types recruit but with different rates	$\lambda_{0}+\lambda_{1}^{s}\cdot i+\lambda_{1}^{ns}\cdot j$	$\lambda_{0}+\lambda_{1}^{s}\cdot i+\lambda_{1}^{ns}\cdot j$	μ*i*	μ*j*
Both T cell types recruit but only their own type	$\lambda_{0}+\lambda_{1}^{s}\cdot i$	$\lambda_{0}+\lambda_{1}^{ns}\cdot j$	μ*i*	μ*j*

*For the general mathematical model describing co-clustering of two cell types (Equations 12–15) we define parameters determining the rate of T cell entry into the cluster ($\lambda_{ij}^{s}$ and $\lambda_{ij}^{ns}$) and the rate of exit from the cluster ($\mu_{ij}^{s}$ and $\mu_{ij}^{ns}$) where superscripts “s” and “ns” stand for Plasmodium-specific and non-specific T cells, respectively, ij denotes a cluster with i specific and j non-specific T cells around a given liver stage. For example, $\lambda_{ij}^{s}$ denotes the entry rate of specific T cells into a cluster with i specific and j non-specific T cells. Parameters λ_0_ (the initial entry rate), μ (per capita exit rate), and λ_1_ (increase in entry rate with cluster size) are found by fitting the numerical solution of the mathematical model [given in Equations (12–15)] to the co-clustering data (dataset #3)*.

#### 2.2.5. Stochastic Simulations

We simulated cluster formation using the Gillespie algorithm as previously described (44). In short, for every iteration we first determined randomly the time of the change in cluster size which is determined by the total rate at which clusters could increase or decrease in size (e.g., in the DD recruitment model this rate for a cluster of size *k* is λ_0_ + *kλ*_1_ + *kμ*). The second step was to then choose at random which of two events (cluster size increase or decrease) occurs; this is determined by the relative value of the entry rate into the cluster (e.g., λ_0_ + *kλ*_1_) or exit from the cluster (e.g., *kμ*).

#### 2.2.6. Statistics

Our clustering data are given as the number of T cells found in the 40 μm radius from a given parasite following intravital microscopy imaging (29), i.e., the data are simply a column of integers representing T cell numbers per parasite (in co-clustering experiments the data also represent the number of Plasmodium-specific and non-specific T cells found per parasite). As the data shows, in many cases the majority of parasites have no T cells associated with them within a few hours after T cell transfer (29, and see Results section).

To estimate parameters of mathematical models we used a likelihood approach where the likelihood represents the product of probabilities to observe clusters of different sizes

(16) $$\begin{array}{l} L(parameters|data)=P(data|parameters)=\prod_{k=0}^{k_{\max}}{\left(P_{k}\right)}^{x(k)}, \end{array}$$

where *P*_*k*_ is the mathematical model-predicted probability of observing a cluster of size *k* according to a set of parameter values, *x*(*k*) is the number of clusters of size *k* in the data, and *k*_max_ is the maximal cluster size in the data. In this procedure, the probability *P*_*k*_(*t*) can be either given analytically as a steady state solution [e.g., Equation (4)] or can be found by numerically solving the basic mathematical model predicting *P*_*k*_(*t*) at a particular time [e.g., Equations (1,2)]. When fitting numerical solutions of the model to experimental data in some cases we fixed the rate of exit of T cells from clusters μ to different values because we found that it is generally impossible to accurately estimate both entry and exit rates simultaneously (see Results section).

The models were fitted by calculating negative log-likelihood L=-logL and using routine FindMinimum in Mathematica version 11. When alternative models were fitted to the same dataset, we compared quality of the model fits to the data by comparing Akaike weights *w* based on the corrected Akaike Information Criterion (AIC) (45):

(17) $$\begin{array}{l} \text{AIC}=-2\log L+2p+\frac{2p\left(p+1\right)}{N-p-1}, \end{array}$$

where *p* is the number of model parameters and *N* is the number of data points (parasites). AIC provides a score for each model based on its maximum likelihood value and the number of model parameters. The model with the lowest AIC score is considered to be best relative to the tested models. Weights of a given model can be treated as a likelihood of the model in the list of the tested models. As a rule of thumb, models with *w* < 0.05 can be considered to be inconsistent with experimental data in favor for models with higher weights. In addition, when comparing nested models, where one model is a special case of another, we used the likelihood ratio test. For most of our analyses 95% confidence intervals (CIs) in parameter estimates were calculated by bootstrapping the data on cluster size for individual parasites with replacement with 1,000 simulations (46). In some cases, for example, when fitting co-clustering mathematical model to data, individual fits were slow and it was not feasible to perform confidence interval estimation using bootstrap. Instead, we used profile likelihood to estimate CIs (47, 48).

## 3. Results

### 3.1. Clustering of Endogenous CD8 T Cells Does Not Allow to Discriminate Between T-Cell-Intrinsic and T-Cell-Extrinsic Models of Cluster Formation

Our analyses in Cockburn et al. (29) attempted to explain mechanisms behind the formation of clusters around Plasmodium liver stages from the T-cell-centric point of view; namely, we assumed that cluster formation is dependent on the presence of T cells [e.g., DD recruitment model, see Equation (6)]. However, it is possible that a very different alternative mechanism drives the formation of clusters, which is T-cell-extrinsic. In this case, variable clustering of T cells around the liver stages is driven by variability in the environment, for example, due to the level of “attractiveness” of individual parasites. This may arise because individual parasites may induce different degrees of inflammation as they migrate from the blood into the liver parenchyma, or some parasites may infect hepatocytes which are located in liver parts with a larger blood flow, increasing the chance of T cells to locate such parasites (35, 49–51).

**Figure 3:**
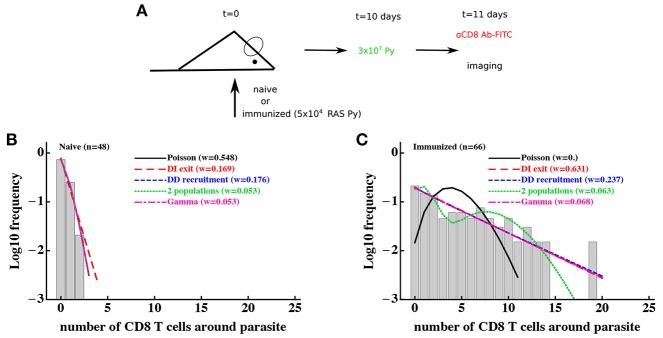
Models assuming time-invariant but spatially-variable environment are consistent with the data on clustering of CD8 T cells in mice immunized with radiation-attenuated sporozoites (RAS). **(A)** Mice were immunized with 5 × 10^4^ Py RAS or left unimmunized. Ten days later, mice were infected with 3 × 10^7^ wild-type Py, expressing GFP. One day later CD8 T cells were labeled with 4 μg PE-conjugated anti-CD8 mAbs and clustering of CD8 T cells around Py-infected hepatocytes in the liver was imaged using intravital microscopy (29). In total 48 (in naive mice, **B**) and 66 (in RAS-immunized mice, **C**) parasites were randomly chosen and the number of T cells in a 40 μm radius were counted. Five different mathematical models were fitted independently to the data on T cell clustering in naive and immunized mice, and the quality of the model fits was evaluated using Akaike weights (*w*). Clustering in naive mice is most consistent with the Poisson (random entry/exit) model, while in RAS-immunized mice models assuming constant environment (“2 populations,” “gamma,” or random birth/death models) fit the data worse than other models, in part due to a larger number of parameters than in the DD recruitment or DI exit models. Parameter estimates of the best fit model and 95% CIs in panel B are θ_0_ = 0.29 (0.17 − 0.42) (Poisson model) and in **(C)** are θ_0_ = 0.81 (0.77 − 0.84) (DI exit model) or θ_0_ = 0.80 (0.52 − 1.16) and θ_1_ = 0.82 (0.73 − 0.88) (DD recruitment model). According to the DD recruitment model, the average cluster size in RAS-immunized mice at steady state **(B)** is 〈*k*〉^*^ ≈ 4.2.

To investigate whether a T-cell-extrinsic mechanism can be sufficient to explain the formation of clusters in our experiments we formulated two alternative mathematical models predicting the formation of clusters of different sizes: in the first model we assumed that there are two populations of parasites with different levels of attractiveness/rate of entry λ_01_ and λ_02_ [“2 population” model, Figure 2D and Equation (10)], and in the second model we assumed that there is a distribution in the level of attractiveness of parasites given by a continuous Gamma distribution [“gamma” model, Figure 2E and Equation (11)]. To test whether models assuming T-cell-intrinsic or T-cell-extrinsic mechanisms of cluster formation perform better, we fitted the models to previously published data on clustering of endogenous CD8 T cells around Py liver stages in mice (29). In these experiments, mice were left naive or were immunized with radiation-attenuated sporozoites (RAS) and then 10 days later infected with wild-type Py expressing GFP (Figure 3A). Clustering of CD8 T cells around GFP-expressing liver stages was visualized by injecting CD8-binding antibody. We fitted five mathematical models to these data using a likelihood approach (Equation 16). This analysis showed that all mathematical models could accurately describe the lack of formation of large (*k* > 5) clusters around Plasmodium liver stages in naive (unimmunized) mice, and the simplest (null) random entry/exit model was favored by the Akaike weights (Figure 3B). While all models provided similar likelihood of the model given the data, lower weights for 2 population and gamma models was due to a larger number of fitted parameters (3 in 2 population/gamma models vs. 1 in the Poisson model).

Consistent with our previous analysis (29), the random entry/exit (Poisson) model could not adequately describe the distribution of cluster sizes in RAS-immunized mice (Figure 3C). Interestingly, both 2 population and gamma models did not provide an improved fit of these clustering data as compared to DD recruitment or DI exit models which fitted the data with similar quality (Figure 3C). This was surprising given that the DD recruitment and DI exit models were not able to accurately describe the two peaks in the cluster size distribution (at 0 and 7 T cells/parasite). A closer inspection revealed that the DD recruitment, DI exit, 2 population, and gamma models provided fits of nearly identical quality as based on the negative log-likelihood values (L≈168), and lower weights were selected for models with more parameters. All models except the Poisson and 2 population models could accurately describe the data (based on goodness-of-fit χ^2^ test); the 2 population model deviation was due to its inability to accurately predict the formation of one cluster with 19 cells (results not shown). Importantly, the 2 population and gamma models could fit other clustering data relatively well, e.g., data on clustering of PyTCR cells or PyTCR cells treated with PT (Figure S1, results not shown). Taken together, these results demonstrate that some clustering datasets do not allow to discriminate between T-cell-intrinsic and T-cell-extrinsic mechanisms of formation of CD8 T cell clusters around Plasmodium liver stages.

### 3.2. Environmental Variability Is Not the Main Driver of Cluster Formation

**Figure 4:**
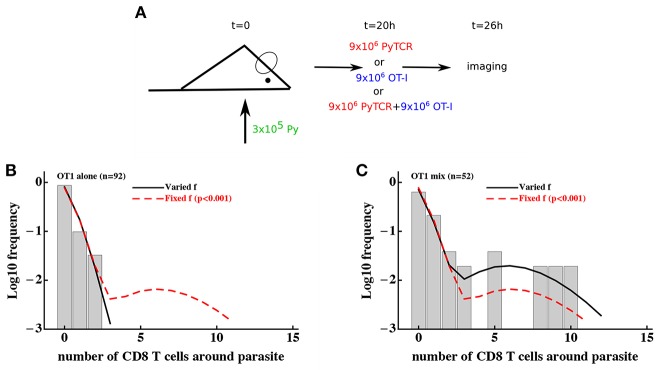
Models assuming time-invariant but spatially-variable environment are unable to accurately describe the clustering of T cells of irrelevant specificity in different conditions. **(A)** Mice were infected with 3 × 10^5^ GFP-expressing Py sporozoites. Twenty hours later 9 × 10^6^ Py-specific activated CD8 T cells (PyTCR), 9 × 10^6^ OT1 T cells (specific to chicken ovalbumin), or mixture of 9 × 10^6^ PyTCR and 9 × 10^6^ OT1 T cells were transferred into infected mice and livers of these mice were imaged using intravital microscopy 6 h later. In total 92 (mice receiving only OT1 cells, **B**) and 52 (in mice receiving a mix of PyTCR and OT1 cells, **C**) parasites were randomly chosen and the number of T cells in a 40 μm radius were counted (29). The “two population” mathematical model (Equation 10) was fitted to these two datasets simultaneously assuming two different entry rates θ_01_ and θ_02_ and either allowing the fraction of attracting parasites *f* to vary between the datasets (solid line) or to be fixed between the datasets (dashed line). Fixing the fraction *f* between the datasets significantly reduced the quality of the model fit of the data as compared to the model in which *f* could vary (likelihood ratio test, $\chi_{1}^{2}=12.4$, *p* < 0.001).

To discriminate between T-cell-intrinsic and T-cell-extrinsic mechanisms of formation of CD8 T cell clusters around Plasmodium liver stages we turned to additional experimental data generated previously (29). In these experiments, Py-specific T cells and T cells of irrelevant specificity (OT1) were transferred either separately or together into mice previously infected with Py-GFP, and the formation of clusters around Py liver stages was measured by intravital microscopy (Figure 4A). We have previously shown that the DD recruitment model describes best (based on Akaike weights) the data on the clustering of PyTCR cells when transferred alone or data on the clustering of PyTCR and OT1 cells when transferred together (29). In contrast, the clustering of OT1 cells alone was best described by the random entry/exit model (29, Figures 4B,C). Therefore, these data indicate that the clustering of T cells, which are not specific to Plasmodium depends on the presence of Py-specific T cells suggesting that variability in parasite's “attractiveness” alone cannot explain these data. We formally tested if the 2 population or gamma models can describe the clustering data of OT1 cells in the following way. We fitted the 2 population model to the data on clustering of OT1 cells alone or in the mixture with PyTCR cells simultaneously. We therefore fitted the models by allowing all three parameters of the model [θ_01_, θ_01_, and *f*, see Equation (10)] to be different for the two datasets or by allowing only the fraction of parasites with different attractiveness level *f* to vary between two datasets while keeping other parameters the same between datasets (Figures 4B,C). In this way, we tested the hypothesis that the clustering of OT1 cells is driven exclusively by factors which are independent of Py-specific CD8 T cells. Because two fits are from nested models, comparing the quality of the fits revealed that the model assuming PyTCR-cell-independent environment fits the two datasets significantly worse ($\chi_{1}^{2}=12.4$, *p* < 0.001). Fitting the gamma model to the same two datasets assuming either identical or variable parameters between the two datasets also suggested that the model with constant parameters fits the data significantly worse (results not shown). Thus, these results strongly suggest that the T-cell-extrinsic models of cluster formation are not consistent with the data on different clustering patterns of OT1 cells in the absence or presence of Py-specific T cells.

It is important to note that the use a specific mathematical model (e.g., 2 population model) simply allows to formally test if distributions of cluster sizes of OT1 cells are different in two different conditions. This can be also done using a χ^2^ test (52) which showed that these distributions are only marginally different ($\chi_{8}^{2}=16.1$, *p* = 0.04). Thus, the use of models allows to obtain much stronger statistical power at falsifying the T-cell-independent (“environment”) hypothesis as the sufficient mechanism of cluster formation.

### 3.3. Several Alternative Retention Models Poorly Describe Data on Clustering of PyTCR Cells

While our experiments of clustering of OT1 T cells either alone or in presence of PyTCR T cells argue against T-cell-extrinsic clustering model, they do not allow to fully discriminate between alternative T-cell-intrinsic clustering models. Fitting the steady state prediction of the DI exit and DD recruitment model to clustering of PyTCR T cells (Figure S1) or clustering of OT1 T cells in the presence of PyTCR cells (29) favored the DD recruitment model (based on Akaike weights). However, it is possible that a specific form of the retention model, i.e., that the per capita exit rate is inversely proportional to the cluster size, was an accidentally poor choice. Therefore, we tested two alternative models of how exit rate from a cluster could depend on cluster size with $\mu_{k}=k^{\alpha }\mu$ or $\mu_{k}=k\mu {\text{e}}^{-\alpha k}$. We fitted the numerical solution of the basic mathematical model (Equations 1, 2) to the clustering of PyTCR T cells (Figure S1A) using a likelihood approach. Both alternative retention models still described the data worse than the DD recruitment model (*w* < 0.001 and *w* = 0.02 for the two models, respectively, results not shown) suggesting limited support for the hypothesis that retention of T cells plays the major role in cluster formation. Therefore, in our following analyses we focus exclusively on the DD recruitment model.

### 3.4. No Evidence That CD8 T Cells of Irrelevant Specificity Influence Clustering

**Figure 5:**
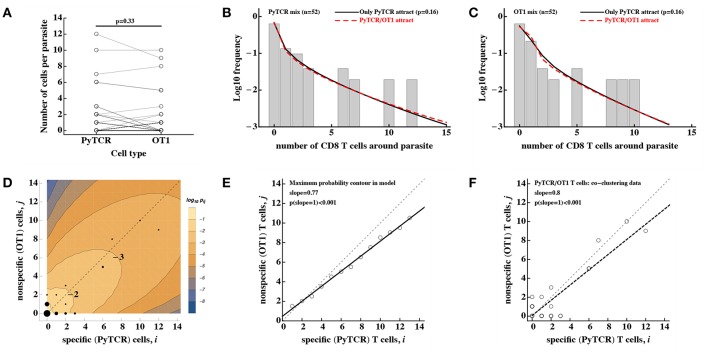
No evidence that activated CD8 T cells of irrelevant specificity play a significant role in cluster formation. Experiments were performed as described in Figure 4A and the number of T cells, specific to Py (PyTCR) and T cells of irrelevant specificity (OT1), found in a 40 μm radius of *n* = 52 randomly chosen parasites in the liver was counted using intravital imaging. **(A)** No difference in the number of Py-specific and non-specific T cells found around Py liver stages (Wilcoxon signed rank test). **(B,C)** We fitted a series of mathematical models assuming how Py-specific or non-specific T cells mediate attraction to the infection site [co-clustering model, Equations (12–15)], and fits of two models where either only PyTCR T cells attract (solid lines, **B,C**) or both PyTCR and OT1 T cells attract (dashed lines, **B,C**) as well as data (bars) are shown. A simpler model in which only PyTCR T cells mediate attraction describes the data as well as the more complex model in which both types of T cells attract to the cluster (likelihood ratio test, $\chi_{1}^{2}=1.95$, *p* = 0.16). Bars in **(B,C)** are the observed frequencies of parasites with a variable number of PyTCR **(B)** or OT1 **(C)** T cells. In **(D)** we show the data (points) and predictions of the model in which only Py-specific T cells attract all activated cells to the infection site (contours); model prediction is the log_10_ *P*_*ij*_(6) where *P*_*ij*_ is the probability to observe *i* Py-specific and *j* non-specific T cells around the parasite at *t* = 6h after T cell transfer. The point size represent the number of parasites having a given number of PyTCR/OT1 cells in 40 μm radius, and thin dashed lines shows the line with slope = 1. In **(E)** points represent the prediction of the model on the number of PyTCR/OT1 T cells in clusters where *P*_*ij*_ reaches maximum for *i* + *j* = const (see the contour plot in **D**). The solid line represents a regression line for the model predictions with slope = 0.76 which is significantly different from 1 (*t*-test, *p* < 0.001) and the dashed line shows the line with slope = 1. In **(F)** points represent co-clustering data of PyTCR and OT1 T cells around parasites (the same data are shown in **D**). Solid line represents a regression line with slope = 0.8 which is significantly different from 1 (*t*-test, *p* < 0.001). Results in **(D–F)** indicate model-predicted slight bias toward clustering of a larger number of PyTCR T cells which is not directly observed in the data **(A)**.

In our previous analysis we showed that the DD recruitment model-based fit of the data on the clustering of PyTCR and OT1 cells in the co-transfer experiments (Figure 4A) predicted similar relative recruitment rate parameters θ_0_ and θ_1_ [see Table S1 in Cockburn et al. (29)]. However, the previous analysis treated clustering of PyTCR and OT1 cells in the co-transfer experiments independently, and here we extend this analysis by considering potential mechanisms behind co-clustering of these two cell populations. First, we found that there was no significant difference in the number of PyTCR or OT1 T cells clusters around Plasmodium liver stages with similar proportions of parasite having more PyTCR or OT1 cells (Figure 5A). To investigate whether the data on the co-clustering of T cells may provide evidence of OT1 T cells assisting in cluster formation we developed a mathematical model tracking the dynamics of co-clustering of two types of cells [see Equations (12–15) in Material and Methods] and fitted that model to the co-clustering data (dataset #3) using a likelihood approach. As we show in the next section, our clustering data do not allow to identify both the rate of T cell entry into the cluster and exit rate from the cluster from measuring clusters at one time point. Therefore, in this analysis we fixed the per capita exit rate μ = 0.5/h and estimated entry rates. Our overall results were robust to several other tested values of the exit rate, such as μ = 0.1/h or μ = 3/h even though estimates of the entry rates were strongly dependent on the assumed exit rate (results not shown and see next section).

Using the DD recruitment model we tested several different mechanisms of how specific and non-specific T cells may participate in cluster formation (see Table 1). Despite the highly correlated numbers of the Py-specific and non-specific T cells around Plasmodium liver stages (Figure 5A), different roles of these two CD8 T cell types seem to be inherent in the data (Table 2). Specifically, the model in which PyTCR T cells attract all cells into the cluster was statistically better at describing these data as compared to any other model tested (based on Akaike weights); interestingly, an alternative model in which OT1 cells exclusively drive cluster formation could not fit the co-clustering data well (model “Only OT1 cells recruit” in Table 2).

**Table 2:** Comparing alternative models which assume different contributions of Py-specific (PyTCR) and non-specific (OT1) T cells to cluster formation.

**Model**	**λ_0_, *1/h***	**λ_1_, *1/h***	**L**	**AIC**	***w***
Only PyTCR cells recruit	0.14 (0.1, 0.19)	0.58 (0.46, 0.74)	128.0	260.3	**0.40**
Only OT1 cells recruit	0.15 (0.11, 0.21)	0.60 (0.47, 0.76)	131.4	267.8	0.01
PyTCR and OT1 cells recruit at the same rate	0.12 (0.085, 0.17)	0.32 (0.26, 0.39)	130.7	265.6	0.03
PyTCR and OT1 cells recruit at different rates	0.17 (0.13, 0.23)	PyTCR = 0.67 (0.54, 0.85), OT1 = −0.16 (−0.03, −0.22)	127.1	260.6	0.34
PyTCR and OT1 cells recruit at different rates toward different cell types	0.18 (0.13, 0.24)	PyTCR:PyTCR = 0.73 (0.58, 0.91), PyTCR:OT1 = 0.61 (0.43, 0.84), OT1:OT1 = −0.15 (−0.05, −0.37), OT1:PyTCR = −0.23 (−0.02, −0.22)	126.7	264.8	0.04

*We fit the basic mathematical model on co-clustering of Plasmodium-specific and non-specific T cells (Equations 12–15) to the data on co-clustering of T cells around Py liver stages assuming DD recruitment model and different mechanisms of how T cells contribute to cluster formation (see Table 1 for tested models). Here we list the estimated initial recruitment rate λ_0_ and how recruitment rate changes with cluster size λ_1_ (i.e., in the DD recruitment model the recruitment rate is λ_k_ = λ_0_+kλ_1_), the negative log-likelihood L, AIC, and Akaike weights w for the model fit. In these fits the total exit rate of T cells from the cluster of size k was fixed to μ_k_ = 0.5k/h. In the column with estimates for λ_1_ we list specifically the predicted change in the cluster “attractiveness” by a given type of T cell (specific or non-specific) and toward a given type of T cells. For instance, an estimate λ_1_ = 0.58/h for the model in which only PyTCR cells recruit assumes that PyTCR cells recruit specific and non-specific T cells at the same rate. In another model notation “PyTCR:OT1” denotes the recruitment rate induced by PyTCR cells for OT1 cells. Numbers in parentheses indicate 95% confidence intervals for parameter estimates. Bold value indicates the weight of the best fit model*.

In two separate models we tested whether OT1 cells “help” in the formation of clusters which is driven by Py-specific T cells. Perhaps unsurprisingly in both models (“PyTCR and OT1 cells recruit at different rates” and “PyTCR and OT1 cells recruit at different rates toward different cell types”) we found no evidence that OT1 cells enhance cluster formation (Table 2 and Figures 5B,C). In contrast, parameter estimates suggested that OT1 cells may inhibit cluster formation because the estimated OT1-driven recruitment rates λ_1_ were negative (Table 2); however, improvements of the fits of these two more complicated models were not supported by the likelihood ratio test (*p* > 0.1, see Figures 5B,C). Thus, our results suggest that non-specific T cells are “passive” participants in the clusters and do not significantly promote or impede the formation of clusters. A similar result was obtained recently using another Plasmodium experimental system (31).

Predictions of our best-fit mathematical model in which only PyTCR cells recruit all activated T cells to the site of infection can be shown as the distribution of cluster sizes for each cell type (e.g., Figures 5B,C) as well as the probability to observe a cluster with *i* PyTCR and *j* OT1 cells (Figure 5D). Careful examination of this fit revealed that the model predicts a slight bias toward having more PyTCR cells per cluster than OT1 cells (Figure 5E). Linear regression analysis of the co-clustering data indeed suggests that there may be bias toward having more PyTCR cells than OT1 cells per cluster (Figure 5F); however, this result is not fully consistent with another analysis (e.g., Figure 5A), and the application of linear regression to data with integers may not fully appropriate. While the existence of such a bias is indeed in line with the analytical analysis of the steady state distribution of cluster sizes (see Supplemental Information for mathematical proof), this bias is small (perhaps one extra PyTCR cell in clusters of a total size of 20), and biological relevance of such a bias for the killing of the parasite is most likely limited.

### 3.5. Clusters of Py-Specific CD8 T Cells Around Py-Infected Hepatocytes Are Formed Rapidly

Our analyses so far made an assumption that clusters around Plasmodium liver stages reach a steady state by 6–24 h after T cell transfer. To understand potential limitations of this approach, we therefore performed several additional analyses.

Because our main mathematical model of T cell clustering Equations (1), (2) can be solved numerically and thus fitted to experimental data assuming a specific clustering mechanism (e.g., DD recruitment model), we investigated if the rates of T cell entry into the cluster (λ_0_ and λ_1_) and rates of exit from the cluster (μ) can be estimated from data in which PyTCR cell clusters around Py-infected hepatocytes were observed at 6 h after T cell transfer. Interestingly, fitting the DD recruitment model Equations (1), (2) to data on the clustering of PyTCR cells transferred alone (Figure S1B or Figure 5A) revealed that model fits favored very high entry and exit rates, e.g., rates exceeding 20–30/h (results not shown). By fixing the exit rate from the cluster to multiple values we found that estimates of the absolute and relative values of the entry rate depended strongly on the exit rate values, and the relative entry rates (θ_0_ and θ_1_) approached constant values at high exit rates (Table 3). Importantly, all the fits of models with dramatically different exit rates were of nearly identical quality as based on negative log-likelihood suggesting that data on clustering of T cells at one time point are not sufficient to estimate entry and exit rates simultaneously. These results were confirmed for two independent datasets (experiments with PyTCR cells alone as shown in Figure S1A and Figure 5A) although exact values of parameter estimates, such as λ_0_ did slightly vary between two sets of experiments [see Figure S1 and estimates in Table S1 in (29)].

**Table 3:** Estimated relative entry rates in the DD recruitment model (θ_0_ and θ_1_) strongly depend on the value of assumed exit rate from the cluster μ.

	****μ**=0.06/h**	****μ**=0.1/h**	****μ**=0.3/h**	****μ**=1/h**	****μ**=3/h**
Estimated **θ**_0_	1.45 (1.10–1.88)	0.92 (0.70–1.23)	0.41 (0.30–0.51)	0.24 (0.17–0.31)	0.20 (0.14–0.28)
Estimated **θ**_1_	6.31 (4.37–7.71)	4.02 (2.77–4.91)	1.77 (1.29–2.09)	1.04 (0.84–1.17)	0.89 (0.77–0.98)

*We fixed the value of the rate of exit of T cells from the cluster μ to different values [indicated in the top row) and fitted the DD recruitment model (with λ_k_ = λ_0_+λ_1_k and μ_k_ = kμ, see Equations (1), (2)] to experimental data on clustering of PyTCR T cells (n = 130) when transferred alone (cluster formation was observed 6 h after T cell transfer, see Figure 5A). The quality of model fits to data as judged by the negative log-likelihood were nearly identical between different fits (L~194); the fit of the model with μ = 3/h is shown in our previous publication [see Figure S1A and Table S1 in Cockburn et al. (29)]. To compare parameter estimates we show relative entry rates θ_0_ = λ_0_/μ and θ_1_ = λ_1_/μ. In parentheses we shown 95% confidence intervals for parameter estimates. Interestingly, the ratio θ_1_/θ_0_ was relatively constant for different fits (53, and manuscript in preparation)*.

**Figure 6:**
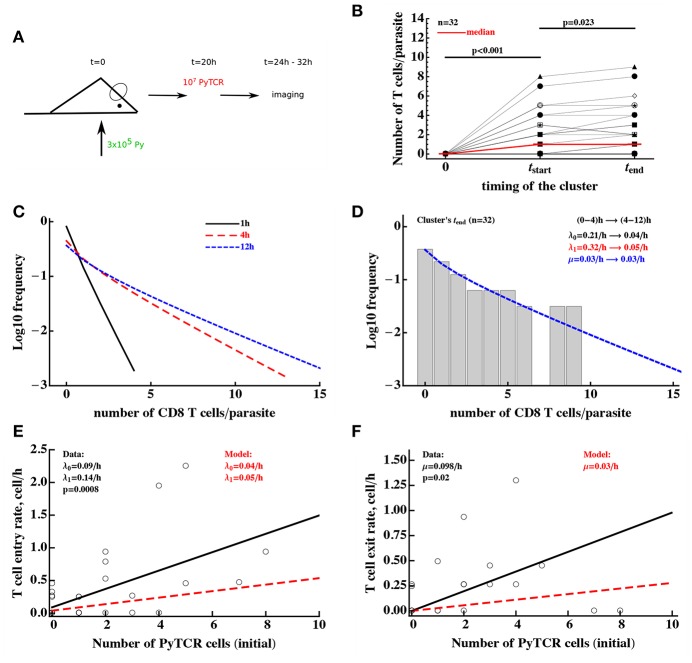
Clusters of CD8 T cells around the parasite are largely formed by 4 h post-T cell transfer. **(A)** Mice were infected with 3 × 10^5^ GFP-expressing Py sporozoites. Twenty hours later 10^7^ Py-specific activated CD8 T cells (PyTCR) were transferred into infected mice and livers of these mice were imaged using intravital microscopy between 4 and 12 h later. In total 32 parasites were randomly chosen and number of T cells in 40 μm radius of the same parasites were counted at both times (29). **(B)** Significant increase in the median size of the cluster around Py-infected hepatocytes was observed in the first time period and there was a moderate increase in the median cluster size in the following 4–8 h (Wilcoxon sum rank test). Thick red line shows change in the median number of T cells per parasite. In these experiments, 44 and 38% of all parasites did not have a single CD8 T cell nearby for first and last measurement of T cell clusters, respectively. **(C)** We plot the distribution of cluster sizes as predicted by the best fit model at different times after T cell cluster 4. The best fit model was a model assuming DD recruitment Equations (1), (2) with entry rates into the cluster being dependent on the time period (0–4 and 4–12 h) but with the same exit rate during 12 h period. Estimated parameters and their 95% confidence intervals for 0–4 h time interval are λ_0_ = 0.21 (0.11 − 0.34)/h and λ_1_ = 0.32 (0.11 − 0.49)/h; for 4-12h time interval are λ_0_ = 0.04 (0.0 − 0.10)/h and λ_1_ = 0.05 (0.02 − 0.08)/h with the exit rate μ = 0.030 (0.0 − 0.086)/h. **(D)** We show the observed distribution of cluster sizes at the last measurement for each parasite and predictions of the DD recruitment model for 12 h after T cell transfer. **(E,F)** Correlation between the T cell entry rate into the cluster **(E)** or exit rate from the cluster **(F)** as the function of the initial number of PyTCR T cells in the cluster. Points are experimentally measured values from Cockburn et al. (29), solid lines show the regression lines with estimated intercept λ_0_ = 0.09/h and slope λ_1_ = 0.14/h **(E)** or slope μ = 0.098/h **(F)**; both slopes are significantly different from zero (*t*-test). Dashed lines in **(E,F)** show prediction of the mathematical model for the recruitment and exit rates estimated by fitting DD recruitment model to the clustering data.

To gain further insights into the kinetics of T cell cluster formation we analyzed additional data in which the same parasites (*n* = 32) were followed after T cell transfer over time and cluster sizes at different time points were recorded (Figure 6A and Figure S2). In these experiments imaging started between 4 and 8 h after T cell transfer and followed for about 4 h (29). As expected there was a variable and statistically significant increase in the number of T cells found around individual Py-infected hepatocytes between T cell transfer and start of imaging (*t*_start_, Figure 6B). In contrast, in the following ~2–8 h there was a minor change in cluster sizes (*t*_end_, Figure 6B). However, because imaging of CD8 T cell cluster formation started at different time points after T cell transfer there may be biases associated with the simple analysis of the data which takes into account only start and end time points of the clusters (e.g., Figure 6B). To obtained more accurate insights we further analyzed these data using mathematical models of cluster formation.

To take full advantage of these “longitudinal” data in which T cell cluster formation was followed over time for individual parasites (Figure S2), we divided the data into individual “paths,” i.e., the number of T cells found near the parasite at sequential time points. For example, a parasite which did not have any T cells nearby and for which measurements were done at 0, 4, and 6.2 h after T cell transfer, the path is “0 → 0 → 0.” For the parasite that was surrounded by most T cells in these experiments, the path is “0 → 8 → 9” for times 0, 4, and 6.2 h post-T cell transfer (Figure 6B). A mathematical model of cluster formation can then be used to calculate the likelihood of a particular path by assuming that individual “sub-paths” along the path are independent (and thus by multiplying likelihoods of the model for individual sub-paths). For example, the probability to observe the path “0 → 8 → 9” at times (0, 4, 6.2)h is simply the product of the probability to observe 9 T cells in the cluster at 6.2 h given that at 4 h there were 8 T cells in the cluster and the probability to observe 8 T cells in the cluster at 4 h given that at 0 h there were 0 T cells in the cluster:

(18) $$\begin{array}{l} p_{\text{path}}=P_{9}\left(6.2|k=8,t=4\right)\times P_{8}\left(4|k=0,t=0\right), \end{array}$$

where the probability *P*_*k*_(*t*|*i, t*_0_) was calculated using the basic model [see Equations (1), (2)] with initial conditions *P*_*i*_(*t*_0_) = θ_*ij*_ and θ_*ij*_ is Kronicker delta (θ_*ij*_ = 1 if *i* = *j* and θ_*ij*_ = 0, otherwise). Fitting the DD recruitment model to these “longitudinal” data subdivided into “paths” resulted in the following entry/exit rates λ_0_ = 0.14/h, λ_1_ = 0.16/h, μ = 0.09/h. Additional analysis by fixing exit rate μ to different values and then comparing quality of the model fit using likelihood ratio test revealed that estimate of the parameters are relatively robust (i.e., fixing the exit rate to much lower or much higher values resulted in fits lower quality as judged by likelihood ratio test). Furthermore, by resampling the paths with replacement we found relatively small confidence intervals for the estimated parameters suggesting that measurement of T cell clusters longitudinally allows for a relatively accurate estimates of all three parameters of the DD recruitment model determining the kinetics of cluster formation (results not shown).

Parameter estimates of the model fitted to “longitudinal” (paths) data suggest that rates of entry into the cluster and exit from the cluster are relatively small, and this appears to contradict the formation of relatively large clusters already in 4 h after T cell transfer (Figure 6C). Indeed, model fits did not accurately predict formation of clusters with >5 T cells (results not shown). In addition, while the estimate of θ_0_ = λ_0_/μ was reasonable, the estimate of relative recruitment rate θ_1_ = λ_1_/μ was too low when compared with model estimates for clustering of T cells at 6 h after transfer (e.g., Table 3 for μ = 0.1/h).

The major caveat of this analysis is the assumption that the parameters determining T cell clustering are constant over time. Our data indicate that formation of clusters may be slowing down over time (Figure 6B). Therefore, we fitted the DD recruitment model to the longitudinal/path data assuming that parameters determining kinetics of cluster formation depend on the time since T cell transfer. Given how the data were collected (Figure S2) for our analysis we made the simplest assumption that the rates are constant in two time intervals: (0–4) and (4–12) h but may be different between the time intervals. Assuming that in the DD recruitment model recruitment rates λ_0_ and λ_1_ are time-dependent and the exit rate μ is time-independent, the model fitted the data significantly better than the DD recruitment model with constant parameters (likelihood ratio test, $\chi_{2}^{2}=30.0$, *p* < 10^−6^). Parameter estimates suggest a 6-fold reduction in both λ_0_ and λ_1_ 4 h after T cell transfer (see legend of Figure 6 for actual parameter estimates). A similar decline in both λ_0_ and λ_1_ at 4 h after T cell transfer was confirmed by fitting the model in which both rates declined by the same amount α; such a model fitted the data with a similar quality as the model that allowed for different decline in the two rates with time since T cell transfer (likelihood ratio test, $\chi_{1}^{2}=0.02$, *p* = 0.89). Because the distribution of cluster sizes was measured experimentally at different time points it was not possible to visualize the model fits of the data. However, because model predictions suggested little change in cluster size distributions between 4 and 12 h after T cell transfer (Figure 6C), the model predicted well the distribution of cluster sizes for each of the parasite at the end of imaging (Figure 6D, $\chi_{8}^{2}=1.14$, *p* = 1). Interestingly, this analysis indicated an extremely slow rate of T cell exit from clusters at 4–12 h after T cell transfer suggesting that nearly every cell that enters the cluster after 4 h post-T cell transfer remains in the cluster which is an indirect support for the “retention” model.

An alternative DD recruitment model is one in which recruitment rates into the cluster remain constant over time but exit rates from the cluster change with time. This model did slightly improve the model fit of the data as compared to the model with constant parameters (likelihood change of 3.32, $\chi_{2}^{2}=6.6$, *p* = 0.01) and predicted that constant recruitment rates are λ_0_ = 0.14/h and λ_1_ = 0.19/h, and exit rate for the first 4 h is μ = 0/h and for after 4 h is μ = 0.19/h. This model suggests an alternative interpretation of the cluster formation dynamics—namely that T cells are recruited into the cluster and retained during the first 4 h after T cell transfer—but after the initial time additional recruited T cells have a high chance of leaving. Because the quality of this model fit of the data was significantly worse than that of the model with time-dependent recruitment rates (ΔAIC = 21 or Akaike weight *w* < 0.001 for time-dependent exit rate model), our data appear to be more consistent with the time-dependent recruitment and constant exit. This suggests that the best explanation of the longitudinal clustering data is that the formation of clusters is driven by the DD recruitment model in which the rate of T cell recruitment into the cluster declines over time. Parameter estimates also suggest that the formation of clusters around Py-infected hepatocytes occurs mainly during the first 4 h after T cell transfer.

The dynamics of change in the number of T cells near the parasite between 4 and 12 h were followed by time-lapse intravital microscopy which allowed to calculate the number of T cells entering the cluster and leaving the cluster in this time period (29, Figures 6E,F). Analysis showed that both entry and exit rates were strongly dependent on the cluster size *k* even though there was large variability in the number of T cells entering and exiting individual clusters. Interestingly, the slopes of the dependence of recruitment and exit rates was 2- to 3-fold higher for experimentally measured rates as compared to the parameters found by fitting DD recruitment model to longitudinal data (Figures 6E,F). One potential explanation of this difference is that perhaps not all cells that come near the parasite (i.e., within 40 μm distance) recognize the infection and leave, thus, increasing the overall observed T cell surveillance rate. In contrast, the model only accounts for T cells which actually formed clusters and thus most likely have recognized the parasite.

### 3.6. Density-Dependent Recruitment Model Is Consistent With Akbari et al. (31) Data

Our analysis so far was restricted to data generated in one experimental system in which formation of CD8 T cell clusters was determined following shortly, within 4–6 h, after transfer of activated T cells to mice, previously infected with malaria sporozoites. Recently another experimental set-up was introduced (31). In these experiments, mice first receive *in vitro* activated CD8 T cells; 24 h later mice are infected with Plasmodium berghei sporozoites; then 20 h after infection murine livers are imaged using intravital microscopy (31). We re-analyzed the data from Akbari et al. (31) in which sporozoite-specific (OT-1) and non-specific (2C) CD8 T cells were transferred simultaneously and formation of clusters around Plasmodium-infected hepatocytes was recorded [see Figure 2D in Akbari et al. (31)].

**Figure 7:**
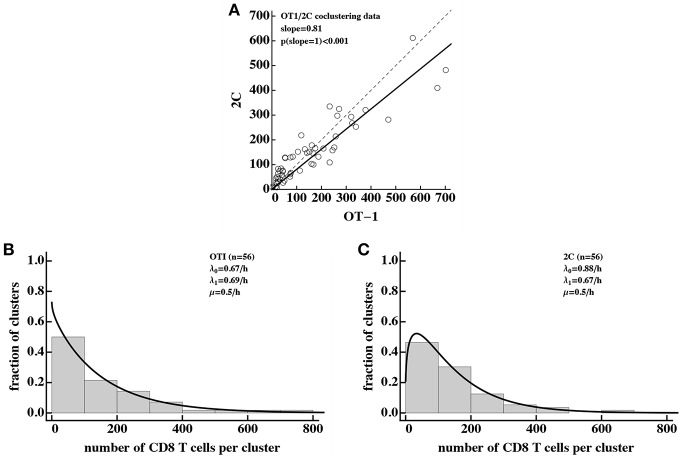
Density-dependent recruitment model is consistent with formation of large clusters. We analyzed the data on co-clustering of Plasmodium berghei (Pb)-specific (OT-1) and control (2C) CD8 T cells from a recently published study (31). **(A)** There is a bias in cluster sizes toward more Pb-specific CD8 T cells per cluster. **(B,C)** The data on cluster distribution and fits of the DD recruitment model assuming that OT-1 **(B)** or 2C **(C)** cluster independently. In fits we fixed the exit rate from the cluster to μ = 0.5*k*/h. Because the experimental data were sparse and frequency of clusters of a given size were the same for different clusters, we plotted the data as a histogram by binning the data into bins with the bin size of 100. To visualize the model fits of the data, we therefore multiplied the model predictions in **(B,C)** by 100. Best fit parameters and estimated 95% CIs are for **(B)**: λ_0_ = 0.67 (0.54 − 0.82)/h, λ_1_ = 0.69 (0.67 − 0.71)/h, and for **(C)**: λ_0_ = 0.88 (0.72 − 1.05)/h, λ_1_ = 0.67 (0.65 − 0.68)/h.

Interestingly, our analysis revealed that there is a slight bias in the numbers of Pb-specific (OT-1) CD8 T cells found in the cluster as compared to 2C CD8 T cells (Figure 7A) which is consistent with our results (Figure 5F). Because of the large numbers of CD8 T cells found in these clusters, we could not directly fit our model to the co-clustering data (the model will need to have nearly 50,000 equations). Instead we fitted the numerical solution of the DD recruitment model [given in Equations (1), (2) with λ_*k*_ = λ_0_ + λ_1_*k* and μ_*k*_ = μ*k*] to the cluster size distribution for OT-1 and 2C cells independently (Figures 7B,C). The model could fit these data with acceptable quality and predicted small difference in recruitment parameters between Plasmodium-specific and non-specific CD8 T cells. Interestingly, the parameter characterizing amplification of the cluster size λ_1_ was very similar for these data and our estimates from co-clustering experiments (see Table 2) suggesting the cluster formation in two systems may be driven by the same mechanism. However, the initial recruitment rates λ_0_ were much higher in these experiments than in our data, perhaps explaining a difference cluster sizes observed in two studies. Taken together, DD recruitment model was consistent with the data from a set of independent experiments.

## 4. Discussion

Studies from two independent groups showed that activated Plasmodium-specific CD8 T cells form clusters around Plasmodium-infected hepatocytes and that such clusters are correlated with elimination of the Plasmodium liver stages (29–31). Application of mathematical models to data on distribution of the number of Py-specific CD8 T cells around randomly chosen parasites suggested that formation of the clusters is not a random process; the model in which activated T cells of different specificities are attracted at a rate proportional to the number of Py-specific T cells already present near the parasite, described the data with best quality (29). More recent work also suggested that formation of CD8 T cell clusters around Plasmodium-infected hepatocytes depends on CD11c^+^ cells and that activated CD8 T cells, specific to irrelevant antigens, do not appear to play a significant role in protection against Plasmodium challenge (31). We analyzed experimental data with the use of mathematical modeling to provide further insights into potential mechanisms of the formation of clusters around Py-infected hepatocytes.

First, we found that several independent experimental datasets are fully consistent with the model in which variability in the number of activated Py-specific CD8 T cells located near the parasite-infected hepatocytes is driven by variability in the “environment” around the infected hepatocytes, providing indirect support for the T cell-extrinsic mechanism of cluster formation (e.g., Figure 3). These results suggested that data on clustering of Py-specific T cells alone may be insufficient to discriminate between T cell-intrinsic and T cell-extrinsic mechanisms of cluster formation (54). A key experiment, rejecting the “variability of the environment” hypothesis as the sufficient mechanism explaining distribution of cluster sizes is one involving either transfer of only OT1 T cells (which are not specific to Py antigens) or OT1 T cells together with PyTCR T cells—only in the latter case, OT1 T cells form co-clusters with Py-specific T cells [Figure 4 and see (29, 31)]. The mathematical model assuming fixed yet variable (between individual parasites) environment was not able to accurately explain such data (Figure 4). The result, however, does not mean that inflammation is irrelevant for parasite's replication in the liver. In fact, recent work suggested that sporozoite infection of the liver does lead to inflammation (36, 50, 51). Our conclusion may seem to contradict to a recent finding that depletion of CD11c-expressing cells reduces the number of CD8 T cell clusters in murine livers (31). However, depletion of CD11c-expressing cells also results in the reduced numbers of Plasmodium-specific CD8 T cells in the liver, which according the DD recruitment model, can dramatically reduce the numbers of large clusters by reducing entry rates λ_0_ and λ_1_ only 2- to 3-fold. Therefore, the direct impact of CD11c-expressing cells on formation of CD8 T cell clusters in the liver remains to be defined.

Second, while OT1 T cells of irrelevant specificity are found in clusters together with Py-specific CD8 T cells, we found no evidence that OT1 improve cluster formation (Figure 5). If anything, OT1 T cells may in fact reduce the rate of recruitment of other T cells into the cluster as indicated by the negative values for the recruitment rate λ_1_ (Table 2); however, this value was not significantly different from zero. Mathematical modeling also suggested that there may be a slight bias in the clusters to have more Py-specific T cells than T cells of irrelevant specificity per cluster but the biological relevance of such a small bias remains unclear. The limited role of T cells of irrelevant specificity in the formation of T cell clusters in Py-infected mice is consistent with the observation that transfers of large numbers of activated CD8 T cells with irrelevant specificity into Plasmodium-infected mice did not impact efficiency at which parasites were killed by Plasmodium-specific T cells (31). Interestingly, and perhaps surprisingly, this result contradicts a recent observation of suppression of development of T-cell-driven type 1 diabetes by islet—non-specific CD8 T cells (55).

Third, by following longitudinal changes in the number of CD8 T cells around individual parasites over time we found that T cell clusters are formed rather rapidly, at least within the first 4 h after T cell transfer (Figure 6B), and mathematical modeling predicted recruitment of T cells to the parasite and retaining of the T cells in the cluster in that time period. Interestingly, the rates of entry into and exit from the clusters declined after the 4 h 6-fold further supporting the conclusion that clusters are formed rapidly and few cells enter and exit the cluster after 4 h since T cell transfer (Figures 6C,D). Stochastic simulations of the formation of clusters assuming DD recruitment model with different entry/exit rates also suggested that between 4 and 8 h post-T cell transfer, entry and exit rates cannot be large (Figure S3). This is because when these rates are large, changes in the cluster size in the 4–8 h time period are highly variable with some clusters growing in size exponentially while other clusters nearly disappearing (e.g., Figures S3C,F). This, however, was not observed in experimental data (shown by dotted histogram in Figures S3D–F and see Figure 6B and Figure S2). Rapid recruitment of CD8 T cells to the liver stages in the first 4 h after T cell transfer may be the result of the specific experimental set-up as it is expected that immediately after intravenous injection, large numbers of T cells would be passing through the liver increasing chances of T cells finding the infection site (56), and that the number of liver-resident CD8 T cells tends to reach a steady state at 2–3 h after T cell transfer (57, James O'Connor and Ian Cockburn, in preparation).

Interestingly, there was some discrepancy between the estimated rates of T cell entry into the cluster and exit from the cluster measured experimentally and predicted by the model (Figures 6E,F) most likely indicating that not all T cells that were observed to come in close proximity with the parasite recognize it. We also found a strong correlation between experimentally measured rates of T cell entry into and exit from the clusters (Figure S4) which may indicate that in addition to T cell-intrinsic mechanisms of clustering, some parasites may be more “attractive” to T cells. Indeed, none of our tested models could well explain the formation of extremely large T cell clusters around Py-infected hepatocytes (e.g., with 15 or more T cells, see Figure S1B) which could indicate the need for future models to include both DD recruitment and variability in parasite's attractiveness.

In this paper we analyzed a number of different datasets that involve different cell types, different experimental set-ups, and different mice. We found it encouraging that some of these datasets were in a way “consistent.” Specifically, we observed similar clustering of CD8 T cells in naive mice (Figure 3A), PT-treated PyTCR T cells (Figure S1B), or activated OT1 T cells of irrelevant specificity (Figure 4A) and the random entry/exit model described these data with nearly identical parameters (likelihood ratio test, $\chi_{2}^{2}=5.38$, *p* = 0.07). The DD recruitment model could describe the distribution of cluster sizes of PyTCR T cells in three different experiments [Figure S1A and data in (29)] with identical parameters ($\chi_{4}^{2}=3.31$, *p* = 0.51). It is therefore possible to use model averaging to provide even tighter confidence intervals for the estimated parameters (45). However, the clustering of CD8 T cells following immunization with radiation-attenuated sporozoites (Figure 3B) did not match well the clustering of the mixture of PyTCR and OT1 T cells ($\chi_{2}^{2}=12.21$, *p* = 0.002) perhaps highlighting potential differences between active and passive immunizations (the latter involving transfer of pre-activated CD8 T cells).

In multiple analyses we found that a DI exit (retention) model did not describe well the clustering data. However, a poorer fit of the model (as compared to other tested models) does not necessarily falsify a model (54), and additional experiments will be needed to formally rule out this model. Fitting the DI exit model to the “longitudinal” data on change in cluster size around individual parasites (e.g., Figure S2) revealed that this model could not accurately describe the data assuming a constant entry and time-dependent exit rates based on negative log-likelihood of the model (L=100.2 vs. L=83.0 of the DD recruitment model with time-dependent recruitment and constant exit rates, results not shown). In addition, if the rate of T cell exit from the clusters found in the DD recruitment model is constant over the course of the first 12 h since T cell transfer, it would suggest that T cells mostly enter the clusters and rarely exit them (given μ = 0.028/h corresponding to the residence time of T cells in the cluster of about 1/μ≈36h), providing some indirect support for the retention model.

Conversely, our result that the DD recruitment model describes most of the data with best quality does not prove that this model is the true mechanism of the formation of large clusters around Py-infected hepatocytes. Future experiments will have to test the major prediction of the model—that clusters of a large size attract more T cells per unit of time. Such experiments may involve measurement of T cell movements in the liver using intravital microscopy and estimating bias in T cell shift toward the parasite. Indeed, our recent work suggested that there is a bias in PyTCR cell movements toward Py-infected cells (34) but more analyses are needed to evaluate whether such a bias depends on the number of T cells already present at the parasite and whether a small bias is sufficient to explain the formation of larger clusters (with *k*~5−10 of CD8 T cells per parasite) within few hours after T cell transfer. Detecting a bias in T cell movement toward the infection site may be complicated as our current analysis predicts that “attraction” seems to be present only during the first 4 h after T cell transfer (Figures 6C,D).

Comparing the DD recruitment model to the data from independent experiments suggested that the model can well describe clustering of Plasmodium-specific and non-specific CD8 T cells (31, Figure 7). However, there are several differences in experimental design and methods to detect clusters between the studies that caution about direct comparison. Clusters in Akbari et al. (31) did not contain fluorescence signal from the parasite, and indeed, our ongoing experiments suggest that it was impossible to find parasites in 20–24 h after the infection. Timing of when clusters were observed was also different [6 h in our studies vs. 20 h in Akbari et al. (31)]. These differences may have contributed to the difference in predicting cluster size change over time: we predict that cluster size growth slows over time suggesting that large clusters around the live parasite are unlikely to be formed. However, because our clusters have been defined while the parasite was detectable, it is possible that clusters continue to grow after parasite's death. This issue will have to be investigated using mathematical models that include parasite's death and is the topic of our future work.

Our analysis has several potential limitations. The biggest issue is that by using numerical solutions of the DD recruitment model we showed that the distribution of cluster sizes at a single time point does not allow to accurately estimate the rates of T cell entry into the clusters and T cell exit from the clusters, and thus, most of our analysis were restricted to estimating relative recruitment rates. Ongoing analysis based on analytical solutions in Bailey (53) has also demonstrated this point using analytical derivations (manuscript in preparation). While the estimated values of the recruitment rates λ_0_ and λ_1_ in the DD recruitment model directly depend on the assumed exit rate μ (see Table 3) we showed that the likelihood of the model fit to data assuming a steady state or dynamics for clusters at a given exit rate μ were nearly identical strongly suggesting that our results on best fit models obtained assuming steady states are robust. Additional simulations showed that the model predicted distribution of cluster sizes is invariant when the model rates and the observation time are scaled suggesting appropriateness of the steady state approximation (Figure S5). However, the actual values of the entry and exit rates cannot be found with certainty as these depend on the actual value of the assumed exit rate (Table 3).

Another complexity in the analysis comes from our finding that rates of T cell entry into the cluster are time-dependent (Figure 6). To investigate whether this impacts our selection of best fit models assuming steady state solutions we did the following. We fitted the DD recruitment model to the clustering data at one time point by assuming that early recruitment rates λ_0_ and λ_1_ are unknown and that late entry rates are fixed to values found from the analysis of longitudinal data (Figure 6) and that the exit rate μ is constant. Under these minimal assumptions the model fit was of nearly identical quality as the model fit of the data assuming a steady state (results not shown). Therefore, even for time-dependent parameters our results determining which models are not consistent with clustering data remain valid.

For our analysis of cluster size distribution the data were obtained from livers of several (2–4) mice. A more sophisticated approach for analysis of such data could be a mixed-effect approach in which the models are fitted to individual mouse data and variability in parameters for individual mice is described by a distribution (58). Because formation of clusters is a likely stochastic process some variability in cluster size distribution between individual mice is expected. However, in our data we saw a relative small variability in the distribution of cluster sizes between individual mice (e.g., Figure S2) justifying our approach.

An important experimental limitation of our data is the way of how experiments were performed whereby pre-activated CD8 T cells were transferred into mice that had been already infected with Plasmodium sporozoites (e.g., Figure S1A). This sequence of events does not fully match the physiological situation in which activated or memory CD8 T cells are already present at the time of sporozoite challenge. In fact, the rapid predicted decline in the rates of T cell recruitment into clusters with time suggests that it may be an artifact of the experimental system. Whether change in the experimental protocol will lead to support of the same mathematical models of cluster formation remains to be determined (and is the focus of our ongoing experiments and analyses).

Mathematical methodologies used in this work provided deeper understanding of how CD8 T cells form clusters around Plasmodium-infected hepatocytes. While formation of such clusters was a novel observation in malaria infection of the liver, clusters of immune cells have been observed in multiple systems including herpes simplex virus (HSV) (59) and *Mycobacterium tuberculosis* (Mtb) (60). In fact, formation of granulomas in Mtb-infected animals and humans is a classical example of T cell clustering around the infection site. Interestingly, both Mtb-specific CD4 T cells and CD4 T cells of irrelevant specificities were found in granulomas of Mtb-infected monkeys (61) which could be explained by the DD recruitment model extended in this work. Movement of neutrophils toward an injury site may also depend on the number of neutrophils that have already reached the site (62). It may be useful to combine mathematical modeling tools for deeper understanding of the mechanisms of formation of clusters of immune cells in these and other systems.

While our work provides some clarification regarding mechanisms of CD8 T cell cluster formation around Plasmodium-infected hepatocytes, many questions remain. In particular, while clusters appear to be important for the death of the parasite (29, 30), whether clusters of a larger size kill the parasites faster remains unknown. Classical work involving killing of chromium-labeled target cells by cytotoxic T lymphocytes (CTLs) suggested a faster killing of targets bound by multiple CTLs (63), and *in vivo*, death of peptide-pulsed targets is directly proportional to the number of peptide-specific CTLs (33). Recent work also suggested that the probability of death of virus-infected cells in skin *in vivo* was higher when the infected cell was contacted by several antigen-specific CD8 T cells (32). Whether the same relationship holds for CD8 T cells killing Plasmodium parasites in the liver remains to be determined. However, several studies have shown that probability of clearance of i.v. injected Plasmodium sporozoites does depend on the number of vaccine-induced CD8 T cells in the liver (22, 23, 64). However, these previous studies are numerically inconsistent suggesting that either 3 × 10^4^ (22) or 10^6^ (23) memory CD8 T cells in the liver are needed for sterilizing protection. Further work is required to accurately quantify the number of CD8 T cells needed for protection.

Our results suggest that activated CD8 T cells of irrelevant specificities do not play a major role in cluster formation, and elegant experiments demonstrated that large numbers of non-specific CD8 T cells do not impair the ability of Plasmodium-specific CD8 T cells to eliminate the parasites (31). However, the latter result was found by using only two different ratios of Plasmodium-specific and non-specific CD8 T cells and 3 mice per group, so it remains to be determined if competition between such cells for the access to infected cells occurs at higher ratios, e.g., as has been observed in another system (55). In natural settings we do expect that Plasmodium-specific CD8 T cells will be likely outnumbered by memory CD8 T cells specific to other infections, and therefore, deeper understanding of such competition may be of relevance to malaria vaccines, inducing liver-resident memory CD8 T cells for protection (65).

Accumulation of large numbers of CD8 T cells around Plasmodium-infected cells raises an intriguing possibility that parasites may in fact attempt to attract CD8 T cells. While this may be detrimental to an individual parasite, as a population this may give an advantage if attracting many CD8 T cells to one site prevents CD8 T cells from effectively locating parasites at other sites. To cause blood-stage infection, there is a need for only one liver stage to mature and release differentiated parasites (merozoites) into the blood stream. Indeed, it should be noted that in most of our experiments, many of surveyed parasites did not have a T cell nearby at 6–8 h after T cell transfer. Future studies may be needed to investigate whether such a strategy is indeed evolutionarily advantageous.

It remains unclear how relevant our results are for T cell-mediated protection of humans against malaria. Because of the need of imaging i.v. injected sporozoites in the liver, large numbers of parasites must be used. This is in contrast with very few sporozoites that humans are likely to be exposed to when bit by infectious mosquitoes.

Taken together, here we illustrated the power of combining the use of detailed quantitative experimental data with mathematical modeling, and limitations that come from inability to make solid conclusions from extensive yet limited experimental data. The field of immunology will likely benefit from closer collaborations between experimentalists and modelers where experimentalists being involved in data analyses and modeling, and modelers are cooperating with experimentalists in designing experiments to test and potentially falsify alternative mathematical models.

## Data Availability

All datasets generated for this study are included in the manuscript/Supplementary Files.

## Author Contributions

RK, IC, and VG designed the study. RK and VG run most of the analyses and wrote first drafts of the paper. HR confirmed some of the numerical results and provided derivation of the bias in co-clustering of antigen-specific and non-specific CD8 T cells. IC performed the additional data analyses and provided feedback on data interpretation and modeling. The final version of the paper was written by VG but all authors contributed to the writing of the final version of the paper via comments and suggestions.

### Conflict of Interest Statement

The authors declare that the research was conducted in the absence of any commercial or financial relationships that could be construed as a potential conflict of interest.
