# Supplementary material for: Clustering of Activated CD8 T Cells Around Malaria-Infected Hepatocytes Is Rapid and Is Driven by Antigen-Specific Cells
Source: Front Immunol. 2019 Sep 20;10:2153. doi: 10.3389/fimmu.2019.02153 (PMC6764016; doi:10.3389/fimmu.2019.02153)
Supplement: Supplementary file 2 [file Data_Sheet_1.pdf]

Clustering of activated CD8 T cells around malaria-infected hepatocytes is rapid and is driven by antigen-specific cells

Reka K. Kelemen, Harshana Rajakaruna, Ian A. Cockburn, and Vitaly V. Ganusov

Supplemental Information

Clustering of control and pertussis toxin-treated CD8 T cells in the liver

By performing experiments in which either PyTCR cells or PyTCR cells, treated with pertussis toxin (PyTCR+PT cells), were transferred into mice, previously infected with Py, we found that PT-treated PyTCR cells form smaller clusters that untreated T cells ([29, Figure S1]). PT treatment inactivates G-protein-coupled receptors (GPCRs) in cells, and in particular, makes PT-treated cells to be unresponsive to chemokine gradients (Spangrude et al. JI 1985). Fitting three basic mathematical models, predicting steady state distribution of T cells around the liver stages, to the data on clustering of PyTCR cells (see Materials and Methods for more detail on how models were fitted to data) confirmed our previous result that DD recruitment model provided the best fit of clustering data (Figure S1B). The random entry/exit model did not fit these data adequately, while the DI exit model provided a visually reasonable fit which was not supported by the Akaike weights or log-likelihood (result not shown). Interestingly, plotting the data and model fits on the log-scale revealed that even the best fit model did not accurately predict formation of a large cluster with 21 PyTCR cells (Figure S1B). Indeed, comparing the prediction of the DD recruitment model with data using a goodness-of-fit χ^2 test showed that the model described the clusters until size 8 well ($\chi^2_{18} = 7.2$, $p = 0.99$), but failed at describing all clusters including one with 21 cells ($\chi^2_{19} = 51.6$, $p < 0.001$). According to the prediction of the mathematical model, the average cluster size of T cells around the liver stages at the steady state is rather small, $\langle k \rangle^* \approx 1.5$ (see eqn. (9)), because most parasites were not found by T cells within 6 hours.

Importantly, however, all three models could accurately describe the formation of clusters by PT-treated PyTCR cells (Figure S1C) suggesting that the formation of these clusters is most likely due to a random encounter between T cells and the Py-infected hepatocyte. Indeed, the estimated relative entry rate θ_0 was similar for two datasets (see legend of Figure S1) and was only slightly higher than the relative rate estimated in our previous work from other experiments involving co-transfer of PyTCR and OT1 cells [29, see Materials and Methods and below]. Thus, this result further supports the conclusion that GPCR-mediated signaling is important in the formation of CD8 T cell clusters around Plasmodium liver stages.

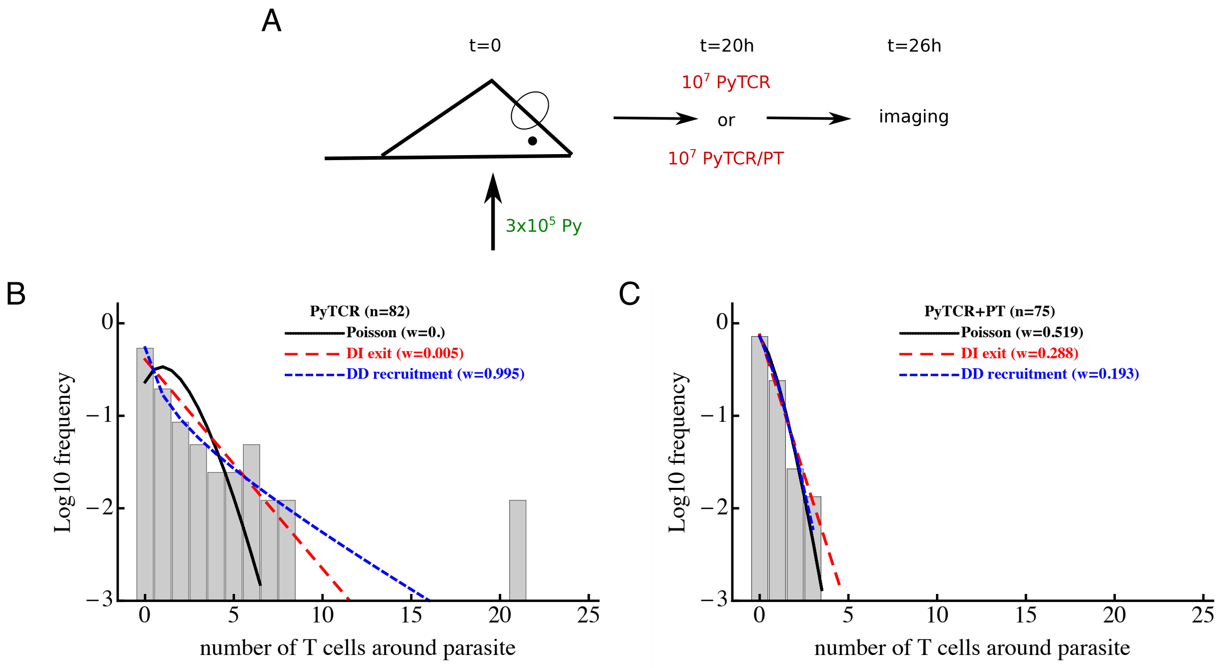

Figure S1: Pertussis toxin (PT)-treated T cells form clusters around Plasmodium yoelii (Py)-infected hepatocytes randomly. Panel A: mice were infected with 3×10^5 GFP-expressing Py sporozoites. Twenty hours later 10^7 Py-specific activated CD8 T cells (PyTCR) or 10^7 PyTCR T cells pretreated for one hour with 1 μg/mL PT were transferred into infected mice and imaged with intravital microscopy 6 hours later [29]. The number of T cells in 40 μm radius of randomly chosen $n = 82$ (PyTCR) or $n = 75$ (PyTCR+PT) parasites was recorded; the frequency of size of such clusters is shown in panels B-C by bars. Panels B and C: we fitted three mathematical models (Poisson (eqn. (4)), DI exit, (eqn. (5)), and DD recruitment (eqn. (6))) to these experimental data using likelihood approach (eqn. (16)) and calculated Akaike weights (w) (see Materials and Methods for more detail). Results suggest that clustering of PyTCR T cells is best described by density-dependent recruitment model while clustering of PT-treated T cells occurs mainly randomly. Parameter estimates of the best fit model and 95% CIs in panel B are $\theta_0 = 0.31$ $(0.20 - 0.48)$ and $\theta_1 = 0.79$ $(0.59 - 0.88)$ (DD recruitment model) and in panel C are $\theta_0 = 0.33$ $(0.21 - 0.48)$ (Poisson model).

Bias in clustering of Plasmodium-specific CD8 T cells and T cells of irrelevant specificity

In fitting mathematical models to data on co-clustering of PyTCR (Py-specific) and OT1 (OVA-specific) CD8 T cells we found slight bias in the number of PyTCR cells in a given cluster. Here we provide mathematical justification of this observation.

Defining the problem. The linear system of ODEs given in eqns. (1)–(2) can be written as follows:

$$\frac{d\vec{P}_{i,j}}{dt} = A(\lambda_{i,j}, \mu_{i,j})\vec{P}_{i,j}(t), \tag{S.1}$$

where $A(\lambda_{i,j}, \mu_{i,j})$ is the probability transition matrix of time-independent entry and exit rate parameters $\lambda_{i,j}$ and $\mu_{i,j}$, respectively, and the subscript (i, j) denotes the number of PyTCR and OT1 cells of a given cluster, respectively, specific to the probabilities and rate parameters. Here, we consider the PyTCR density-dependent recruitment co-cluster model, where we assume that entry rates $\lambda_{i,j}$ of cell combinations (i, j) are time-constant, and both PyTCR and OT1 cells are attracted to a cluster depending on the density of PyTCR cells (type i) in the cluster, which is called the DD recruitment model. Thus, we can write $\lambda_{i,j} = \lambda_0 + i\lambda_1$; where λ_0 and λ_1 are parameters. The exit rate $\mu_{i,j}$ from a cluster are per-capita with respect to the particular cell density, i.e., $\mu_{i,j} = i\mu$ or $\mu_{i,j} = j\mu$, where $\mu > 0$ is a constant.

Defining $\theta_0 = \lambda_0/\mu$, $\theta_1 = \lambda_1/\mu$, we can write

$$\frac{d\vec{P}_{i,j}}{dt} = \mu A(\theta_0, \theta_1)\vec{P}_{i,j}(t). \tag{S.2}$$

Consider the solutions $P^n_{i,j}$ of the above system of equations written in a matrix of size $n \times n \in \Re$, with the largest possible co-cluster size $k_{max} = n$, i.e., $\max(i + j) = n$, where i (i.e., PyTCR cells in a cluster) denotes the rows, and j (i.e., OT1 cells in a cluster) denotes the columns of the matrix. The previous results of fitting DD recruitment model to the data showed that probabilities $P_{i,j}$ of entries (i, j) for smaller j of the upper right triangle of the matrix are likely to be larger than their respective mirror entries (j, i) of the lower left triangle of the matrix, and in contrast, $P_{i,j}$ of entries (i, j) for larger j of the upper right triangle of the matrix are likely to be larger than their respective mirror entries (j, i) of the lower left triangle of the respective matrix. That is, the probabilities for smaller clusters to have a larger number of OT1 cells are greater, whereas the probabilities for larger clusters to have a larger number of PyTCR cells are greater. Here, we show the formation of this systematic bias in the DD recruitment model for $P_{i,j}$ solutions for both the steady state and time-evolving conditions.

Explanation of bias for the system in steady state. Consider the system at steady state, i.e., where the rate of evolution of probabilities of all possible combinations of PyTCR and OT1 cells over time in the ODEs are zero. Thus, we get $A(\theta_0, \theta_1)P_{i,j}(t) = 0$ for $\mu > 0$. Note below that superscript n of $P^n_{i,j}$ indicates the maximum cluster size of the matrix.

I) Case $n = 1$ [i.e., $\max(i + j) = 1$].

The steady state equations of the $n = 2$ matrix system are

$i = \text{PyTCR}$, $j = \text{OT1}$	$j = 0$	$j = 1$
$i = 0$	$P^1_{0,0}$	$P^1_{0,1}$
$i = 1$	$P^1_{1,0}$	

Table S1: Probability table for $n = 1$.

$$P^1_{0,0} = (P^1_{1,0} + P^1_{0,1})/2\theta_0, \tag{S.3}$$

$$P^1_{1,0} = \frac{\theta_0}{[(2\theta_0 + 1) + 2\theta_1]} P^1_{0,0}, \tag{S.4}$$

$$P^1_{0,1} = \frac{\theta_0}{(2\theta_0 + 1)} P^1_{0,0}. \tag{S.5}$$

Since $\theta_0, \theta_1 > 0$, we get $P^1_{1,0} < P^1_{0,1}$. Here, $\sum P^1_{i,j} = 1$ for $i = 0, 1$ and $j = 0, 1$, s.t. $(i + j) \leq 1$. And, the ratio $2\theta_1/\theta_0$ determines how large the $P^1_{1,0} < P^1_{0,1}$ relationship is. Also, note that for any system of equations of size $\max(i + j) = n$ at steady state, the relationship in eq. (S.3) satisfies for probabilities $P^n_{0,0}, P^n_{0,1}, P^n_{1,0}$. That is,

$$P^n_{0,0} = (P^n_{1,0} + P^n_{0,1})/2\theta_0. \tag{S.6}$$

Corollary I: Due to similarity of coeffcients between the linear equations (S.3) and (S.6) of a given system, regardless of the matrix size n, s.t., $\max(i + j) = n$, with fixed rate parameters, $P^n_{0,1}$ and $P^n_{1,0}$ given by eq. ((S.6)) can be expressed as $\alpha_n P^1_{0,1}$ and $\alpha_n P^1_{1,0}$, respectively, for any given $\alpha_n P^1_{0,0}$ of the eq. (S.3), where $\alpha_n \in \Re$. Here, $\alpha_n = 1/\sum P^n_{i,j}$ $\forall (i, j)$ entries.

Thus, the condition $P^n_{1,0} < P^n_{0,1}$ holds true for every solution of a given system of size n.

II) Case $n = 2$ [i.e., $\max(i + j) = 2$].

$i = \text{PyTCR}$, $j = \text{OT1}$	$j = 0$	$j = 1$	$j = 2$
$i = 0$	$P^2_{0,0}$	$P^2_{0,1}$	$P^2_{0,2}$
$i = 1$	$P^2_{1,0}$	$P^2_{1,1}$	
$i = 2$	$P^2_{2,0}$		

Table S2: Probability table for $n = 2$.

Here, we get,

$$P^2_{2,0} = \frac{(\theta_0 + \theta_1)}{(2\theta_0 + 2) + 2(2\theta_1)} P^2_{1,0} \tag{S.7}$$

$$P^2_{0,2} = \frac{\theta_0}{(2\theta_0 + 2)} P^2_{0,1} \tag{S.8}$$

Since we can rewrite $P^2_{1,0} = \alpha_2 P^1_{1,0}$, $P^2_{0,1} = \alpha_2 P^1_{0,1}$ and $P^2_{0,0} = \alpha_2 P^1_{0,0}$, s.t.. $\alpha_2 = 1/\sum P^2_{i,j} \forall (i, j)$ entries, s.t., $\max(i + j) = 2$, from Corollary I, we get

$$P_{2,0}^2 = \alpha_2 \frac{(\theta_0+\theta_1)}{[(2\theta_0+2)+2(2\theta_1)]} \frac{\theta_0}{[(2\theta_0+1)+2\theta_1)]} P_{0,0}^1, \tag{S.9}$$

$$P_{0,2}^2 = \alpha_2 \frac{\theta_0}{(2\theta_0+2)} \frac{\theta_0}{(2\theta_0+1)} P_{0,0}^1. \tag{S.10}$$

Corollary II: If $(x < y)$ and $(\alpha < \beta)$ then $(x+\alpha)/(y+\beta) > (x/y)$ only if $(x/y < \alpha/\beta)$

It follows from Corollary II that in eqns. (S.9) and (S.10), $\frac{\theta_0+\theta_1}{(2\theta_0+2)+2(2\theta_1)} > \frac{\theta_0}{(2\theta_0+2)}$, and also, $\frac{\theta_0}{(2\theta_0+1)+(2\theta_1)} < \frac{\theta_0}{(2\theta_0+1)}$. Thus, whether $P_{2,0}^2 \neq P_{0,2}^2$ is determined by the condition $\frac{\theta_0+\theta_1}{(2\theta_0+2)+2(2\theta_1)} \frac{(2\theta_0+2)}{\theta_0} \neq \frac{\theta_0}{(2\theta_0+1)} \frac{(2\theta_0+1)+2\theta_1}{\theta_0}$, which simplifies to $\frac{1}{4\theta_0} - \theta_0 - 0.25 \neq \theta_1$. Here $\theta_0 < 1$ in general.

Corollary III: It follows from Corollary I, in general, that for a given system of matrix size $n = h+1$, i.e., $\max(i+j) = h+1$, the $P_{k,l}^{h+1}$'s for $\max(i+j) = h-1$ are given as functions of entries $P_{k,l}^h$ s.t., $\max(k+l) = h$, multiplied by the factor α_{h+1}, where $\alpha_{h+1} = 1/\sum P_{i,j}^{h+1} \forall (i,j)$.

$i =$ PyTCR, $j =$ OT1	$j=0$	$j=1$	$j=2$	$\dots$	$j=h-1$	$j=h$
$i=0$	$P_{0,0}^h$	$P_{0,1}^h$	$P_{0,2}^h$	..	$P_{0,h-1}^h$	$P_{0,h}^h$
$i=1$	$P_{1,0}^h$	$P_{1,1}^h$	$P_{1,2}^h$	..	$P_{0,h-1}^h$	
:	:	:	:.	..		
$i=h-1$	$P_{h-1,0}^h$	$P_{h-1,1}^h$				
$i=h$	$P_{h,0}^h$					

Table S3: Probability table for $n = h$.

Thus, for the general case for matrix $\max(i+j) = h$, comparing $P_{h,0}^h$ vs. $P_{0,h}^h$, we get

$$P_{h,0}^h = \alpha_h \frac{(\theta_0+(h-1)\theta_1)}{[(2\theta_0+h)+h(2\theta_1)]} \cdots \frac{(\theta_0+\theta_1)}{[(2\theta_0+2)+2\theta_1)]} \frac{\theta_0}{[(2\theta_0+1)+2\theta_1)]} P_{0,0}^1, \tag{S.11}$$

$$P_{0,h}^h = \alpha_h \frac{\theta_0}{(2\theta_0+h)} \cdots \frac{\theta_0}{(2\theta_0+2)} \frac{\theta_0}{(2\theta_0+1)} P_{0,0}^1, \tag{S.12}$$

for constant α_h. Note that equations (S.11) and (S.12) are functions of $P_{0,0}^1$.

And, thus the $P_{h,0}^h \neq P_{0,h}^h$ holds true depending on the condition if

$$\prod_{m=1}^{h} \left(1+(m-1)\frac{\theta_1}{\theta_0}\right) \neq \prod_{m=1}^{h} \left(1+\frac{\theta_1}{\theta_0/m+0.5}\right) \tag{S.13}$$

as every multiplicative terms, factors after $h > 2$ has a potential to yield $\frac{\theta_0+(h-1)\theta_1}{(2\theta_0+h)+h(2\theta_1)} > \frac{\theta_0}{(2\theta_0+h)}$ for large h, following the Corollary II, for $\frac{(h-1)\theta_1}{h(2\theta_1)} > \frac{\theta_0}{(2\theta_0+h)}$, i.e., $\frac{1}{2} - \frac{1}{2h} > \frac{1}{(2+h/\theta_0)}$ for $\frac{1}{2} - \frac{1}{2h}$ goes from 0

to 1/2, while $\frac{1}{(2+h/\theta_0)}$ goes from 1/2 to zero for h tending from 1 to infinite. In other words, when h gets larger ($h \gg 1$), L.H.S of condition in equation (S.13) turns greater than R.H.S of the condition.

It follows that for small clusters with only one type of cells, the probabilities for the number of OT1 cells to be greater than that of PyTCR cells is greater, i.e., $P^h_{h,0} < P^h_{0,h}$, whereas for large clusters the probabilities for the number of PyTCR cells to be greater than that of OT1 cells is greater, i.e., $P^h_{h,0} > P^h_{0,h}$.

For the case of diagonal entries of matrix size $\max(i+j) = 3$, we can write

$$P^3_{1,1} = \alpha_3 \frac{(\theta_0+\theta_1)P^2_{1,0} + \theta_0 P^2_{0,1}}{(2\theta_0+1)+(2\theta_1)}. \tag{S.14}$$

Here, the proportional contribution of $P^2_{1,0}$ on $P^3_{1,1}$ is greater than that by $P^2_{0,1}$ on $P^3_{1,1}$, whereas, we also get that the proportion of exits from $P^3_{1,1}$ towards $P^2_{1,0}$ is less than that of $P^2_{0,1}$ as $\frac{1}{2\theta_0+1+\theta_1}P^3_{1,1} < \frac{1}{2\theta_0+1}P^3_{1,1}$ from the respective functions of $P^2_{1,0}$ and$P^2_{0,1}$ from steady state equations. Therefore, there is a net differential (flow) of probabilities from PyTCR cells towards the cells with more OT1 cells of the matrix size $n = 3$.

Furthermore, for the general case of the above, i.e., diagonal entries of matrix size $\max(i+j) = 2h+1$ (odd number), we can show that

$$P^{2h+1}_{h,h} = \alpha_{2h+1} \frac{(\theta_0+h\theta_1)P^{2h}_{h,h-1} + (\theta_0+(h-1)\theta_1)P^{2h}_{h-1,h}}{(2\theta_0+2h)+h(2\theta_1)}. \tag{S.15}$$

Here, when $h \to \infty$, the proportional contribution from the entries of $P^{2h}_{h,h-1}$ and $P^{2h}_{h-1,h}$ of matrix $n = 2h$ converges to a single value (constant). The convergence is also true for the proportionate exits from $P^{2h+1}_{h,h}$ into the two entries $P^{2h}_{h,h-1}$ and $P^{2h}_{h-1,h}$. Thus, it follows that when h is larger, the flow of probability from PyTCR cells towards the cells with more OT1 cells converges to zero.

Any probabilities of mirror entries on either side of (h,h) entry of large h, we note that

$$P^{h+1}_{h+1,h} = \alpha_{h+1} \frac{(\theta_0+(h+1)\theta_1)P^h_{h+1,h-1} + (\theta_0+h\theta_1)P^h_{h,h}}{(2\theta_0+2h+1)+(h+1)(2\theta_1)}, \tag{S.16}$$

$$P^{h+1}_{h,h+1} = \alpha_{h+1} \frac{(\theta_0+(h-1)\theta_1)P^h_{h-1,h+1} + (\theta_0+h\theta_1)P^h_{h,h}}{(2\theta_0+2h+1)+h(2\theta_1)}. \tag{S.17}$$

As before, as h increases, the contribution of $P^h_{h+1,h-1}$ towards $P^{h+1}_{h+1,h}$ converges to that of $P^h_{h-1,h+1}$ towards $P^{h+1}_{h,h+1}$ as per the Corollary II. Thus, for large h, $P^{h+1}_{h+1,h} > P^{h+1}_{h,h+1}$ depending on $P^h_{h+1,h-1} > P^h_{h-1,h+1}$, for contributions of $P^h_{h,h}$ towards $P^{h+1}_{h+1,h}$ and $P^{h+1}_{h,h+1}$ converge.

Furthermore, the special case of any two mirror entries $(h,1)$ and $(1,h)$ of matrix size $\max(i+j) = h+1$

$$P^{h+1}_{h,1} = \alpha_{h+1}\frac{(\theta_0 + h\theta_1)P^h_{h,0} + (\theta_0 + (h-1)\theta_1)P^h_{h-1,1}}{(2\theta_0 + h + 1) + h(2\theta_1)}, \tag{S.18}$$

$$P^{h+1}_{1,h} = \alpha_{h+1}\frac{\theta_0 P^h_{0,h} + (\theta_0 + \theta_1)P^h_{1,h-1}}{(2\theta_0 + h + 1) + (2\theta_1)}. \tag{S.19}$$

By Corollary II, here the coeffcients of respective entries in function $P^{h+1}_{h,1}$ are greater than that in the function of $P^{h+1}_{1,h}$ for larger h. Thus, it follows that $P^{h+1}_{h,1} > P^{h+1}_{1,h}$ as $P^h_{h,0} > P^h_{0,h}$ and $P^h_{h-1,1} > P^h_{1,h-1}$ as h gets larger.

For any two mirror entries (i, j) and (j, i) for the case $\max(i + j) = 3$ matrix, we can write

$$P^3_{2,1} = \alpha_3\frac{(\theta_0 + 2\theta_1)P^2_{2,0} + (\theta_0 + \theta_1)P^2_{1,1}}{(2\theta_0 + 3) + 2(2\theta_1)}, \tag{S.20}$$

$$P^3_{1,2} = \alpha_3\frac{(\theta_0)P^2_{0,2} + (\theta_0 + \theta_1)P^2_{1,1}}{(2\theta_0 + 3) + (2\theta_1)}. \tag{S.21}$$

By comparing coefficients of equations (S.20) and (S.21), we note that the contribution of $P^2_{1,1}$ towards $P^3_{1,2}$ is greater than that towards $P^3_{2,1}$. From the same, the contribution of $P^2_{2,0}$ towards $P^3_{2,1}$ is greater than that of $P^2_{0,2}$ towards $P^3_{1,2}$ as per the Corollary II. Thus, if $P^2_{2,0} > P^2_{0,2}$, then $P^3_{2,1} > P^3_{1,2}$, depending on the magnitude of the contribution by $P^2_{1,1}$ towards each, and vice-versa.

For the general case of the above special case, for any (k, l) and (l, k) entries, s.t. $(l + k) = h$, and $l < k$ we get

$$P^h_{k,l} = \alpha_h\frac{(\theta_0 + k\theta_1)P^{h-1}_{k,l-1} + (\theta_0 + (k-1)\theta_1)P^{h-1}_{k-1,l}}{(2\theta_0 + h) + k(2\theta_1)}, \tag{S.22}$$

$$P^h_{l,k} = \alpha_h\frac{(\theta_0 + l\theta_1)P^{h-1}_{l,k-1} + (\theta_0 + (l-1)\theta_1)P^{h-1}_{l-1,k}}{(2\theta_0 + h) + l(2\theta_1)}. \tag{S.23}$$

For $k > l$, using Corollary II, we get that the coefficients of respective entries in $P^h_{k,l}$ are greater than those of mirror entries in $P^h_{l,k}$. Thus, it follows that $P^h_{k,l} > P^h_{l,k}$ if $P^{h-1}_{k-1,l} > P^{h-1}_{l-1,k}$ and $P^{h-1}_{k-1,l} > P^{h-1}_{l-1,k}$ as h gets larger ($h \gg 1$), also because the flow of probability through $P^h_{h,h}$ tends to zero for large h as we have shown above.

Thus, once the probability entries in the lower left triangle, i.e., where PyTCR cell numbers are higher, turn greater than those mirror entries in the upper right triangle, i.e., where OT1 cell numbers are higher, depending on the condition in equation (S.13), and those follow similarly, the pattern of bias remains the same for large h regardless of how large the matrix is.

Explanation of the bias for the system of evolving probabilities over time. The solution to the system at time $t_2 = t_1 + \delta t$, evolved from time t_1 yield $\vec{P}_{i,j}(t_1 + \delta t) = e^{\mu A(\theta_0,\theta_1)\delta t}\vec{P}_{i,j}(t_1)$ where e denotes the matrix exponent with elements $\in \Re$, can be written as $e^{A\delta t} = I + \frac{A\delta t}{1!} + \frac{A^2\delta t^2}{2!} + \ldots$, where I

is the identity matrix. Assuming higher orders (> 1) of the series expansion are negligible compared to its first order solution, we can write the solution to the system, evolved from time t_1 to t_2 as $\vec{P}_{i,j}(t_2) = (I + \mu A(\theta_0, \theta_1)\delta t)P_{i,j}(t_1)$, for elements in A and t are < 1. This yields $\vec{P}_{i,j}(t_1 + \delta t) - \vec{P}_{i,j}(t_1) = \mu A(\theta_0, \theta_1)\delta t P_{i,j}(t_1)$. Note that the steady state solution was obtained by setting this difference to 0. We can write the above as $\frac{\delta \vec{P}_{i,j}(t_1)}{\mu \delta t} = A(\theta_0, \theta_1)\vec{P}_{i,j}(t_1)$.

Thus, the solutions of $\frac{\delta \vec{P}_{i,j}(t_1)}{\mu \delta t}$ of the above to each cluster combination (i, j) as a function of $A(\theta_0, \theta_1)\vec{P}_{i,j}(t_1)$, can be written as, for example from equation (S.3), for the case $n = 1$, $\frac{\delta \vec{P}^1_{0,0}(t_1)}{\mu \delta t} = -P^1_{0,0}(t_1) + \frac{P^1_{1,0}(t_1)+P^1_{0,1}(t_1)}{2\theta_0}$, and similarly from equation 2,$\frac{\delta \vec{P}^1_{1,0}(t_1)}{\mu \delta t} = -P^1_{1,0}(t_1) + \frac{\theta_0 P^1_{0,0}(t_1)}{(2\theta_0+1)+2\theta_1}$, and so on $\forall (i, j)$ combinations given for the case $n = 1$ and so forth for all $n = 2, 3, 4, \ldots$. These can be also written as $P^1_{0,0}(t_1) + \frac{\delta \vec{P}^1_{0,0}(t_1)}{\mu \delta t} = \frac{P^1_{1,0}(t_1)+P^1_{0,1}(t_1)}{2\theta_0}$, and $P^1_{1,0}(t_1) + \frac{\delta \vec{P}^1_{1,0}(t_1)}{\mu \delta t} = \frac{\theta_0}{(2\theta_0+1)+2\theta_1} P^1_{0,0}(t_1)$ and so forth for all $n = 2, 3, 4, \ldots$. Therefore, the patterns in bias, explained under the steady state solutions,$P^n_{i,j}$ in the main text are qualitatively similar for solutions $\left(P^n_{i,j}(t_1) + \frac{\delta P^n_{i,j}(t_1)}{\mu \delta t}\right)$ for all combinations of (i, j), and also for matrices A of any size n. This is because $\left(P^n_{i,j}(t_1) + \frac{\delta P^n_{i,j}(t_1)}{\mu \delta t}\right)$ are functionally equivalent to $P^n_{i,j}$ solutions under the steady sate condition. Thus, the bias discussed in the main text is similar for any $P^n_{i,j}(t_m)$ computed discretely in the form of $P^n_{i,j}(t_2) = P^n_{i,j}(t_1) + \delta \vec{P}^n_{i,j}(t_1)$ step-wise, starting from $t = 0$ to any t, choosing δt, s.t., $\mu \delta t = 1$.

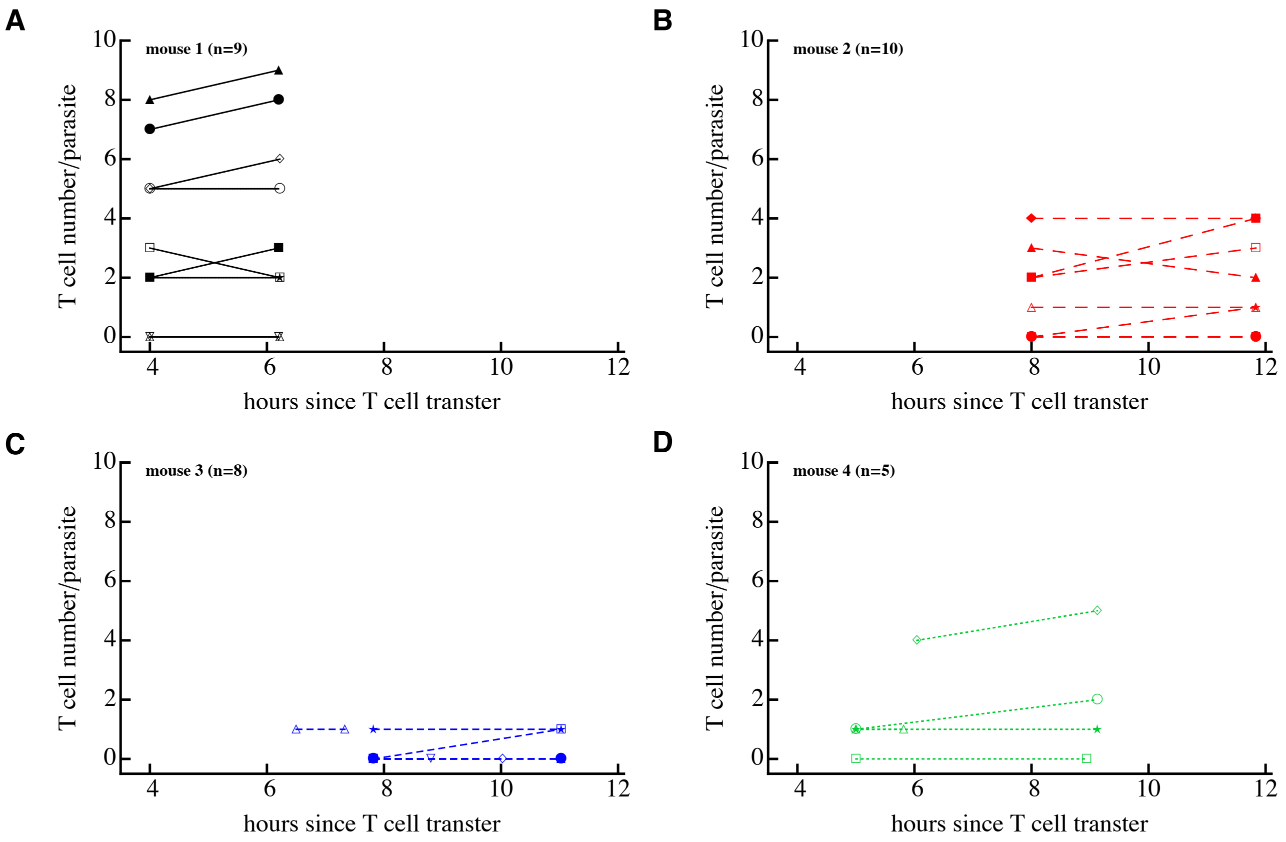

Figure S2: Moderate change in the T cell cluster size over time. We performed imaging experiments as described in Figure 6A and counted the number of T cells found around individual parasites at start and end of intravital imaging done after T cell transfer. Individual panels show change in T cell cluster size around $n = 32$ parasites in four individual mice. Imaging of T cell clusters started at different times in individual mice and followed for different lengths of time. Note that as we observed before, 12 parasites had no T cells near them at both observations. Overall, there was a statistically significant but small change in the cluster size of the imaging period (as summarized in Figure 6B).

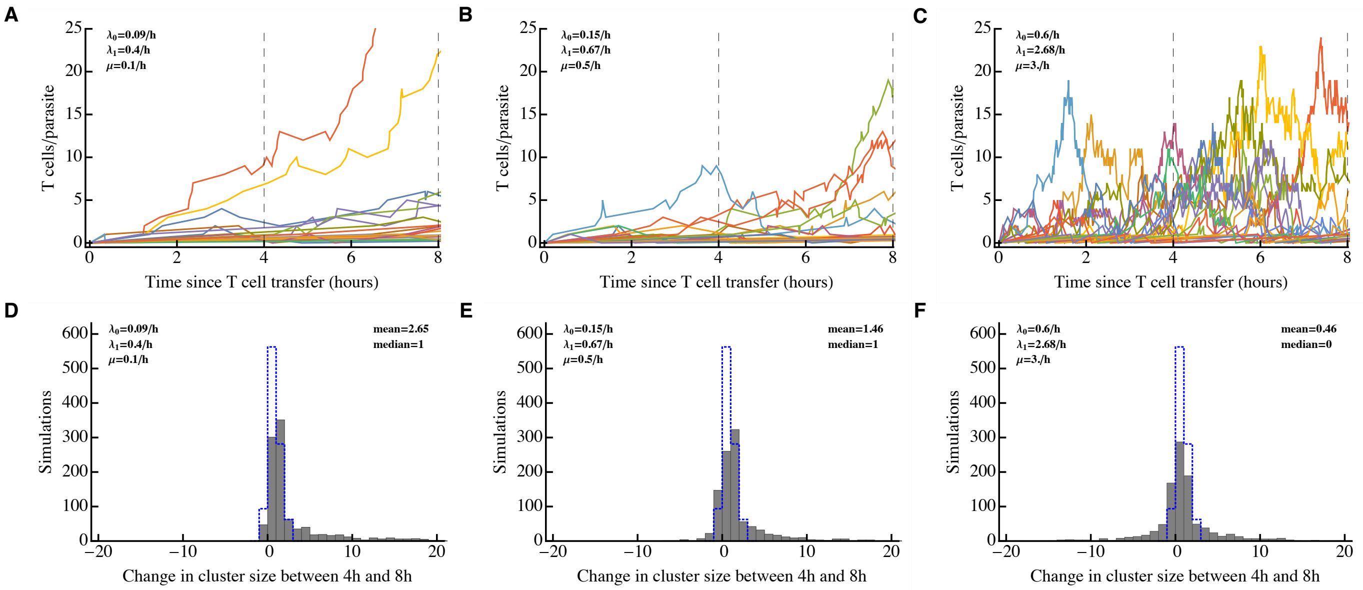

Figure S3: Stochastic simulations of cluster formation suggest an upper limit on the rate of T cell exit from the clusters. We ran Gillespie simulations of the cluster formation assuming different constant (time-independent) values for the entry rates into the cluster (λ_0 and λ_1) and exit rates from the cluster (μ) found by fitting the DD recruitment model to experimental data in Figure 6C-D. Three values of the exit rate were fixed: $\mu = 0.1/\text{h}$ (panels A&D), $\mu = 0.5/\text{h}$ (panels D&E), and $\mu = 3/\text{h}$ (panels C&F) and remaining parameters were estimated by fitting the model (eqns. (1)–(2)) to data (Figure S1B). These parameters are shown on individual panels. We simulated changes in cluster size for $n = 10^3$ parasites. Panels A-C show sample trajectories of cluster sizes of 20 of such simulations, and panels D-F show the change in the size of the cluster between 4 and 8 hours after start of simulation for all simulations (solid bars) or changes in cluster sizes as was observed in experimental data (dotted bars, see also Figure 6B). These simulations indicate that at high exit rates ($\sim \mu = 1 - 3/\text{h}$) and at high entry rates there are large fluctuations in the cluster sizes between 4 and 8 hours (panels C&F) which is not observed in experimental data. Thus, in the 4-8 hour time period exit and entry rates cannot be extremely large for the DD recruitment model to be consistent with experimental data. Furthermore, simulations with smaller rates (panels A&D) also indicate increase in the average cluster size over time (since $\lambda_1 > \mu$) which is also not consistent with the change in cluster size at 4-8 hours post T cell transfer.

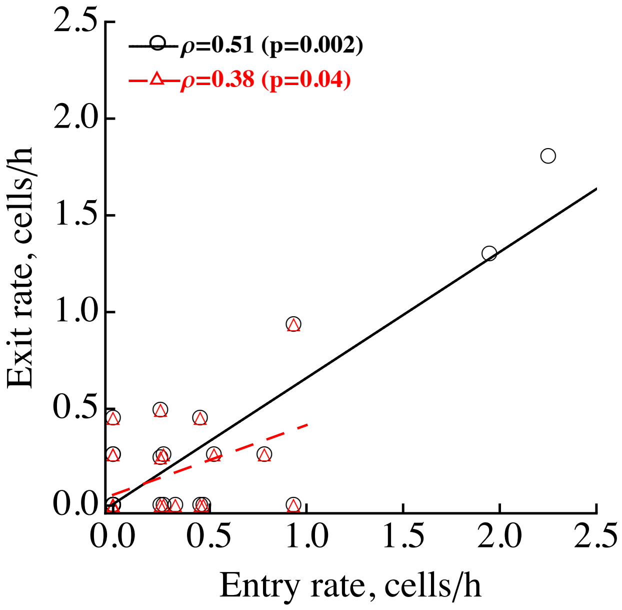

Figure S4: Experimentally measured rate of T cell exit from the cluster correlates with the rate of T cell entry into the cluster. We plotted the correlation between the experimentally measured number of T cells coming with a 40 μm radius of a given parasite per unit of time (entry rate, see Figure 6E) and the number of T cells leaving a given cluster per unit of time (exit rate, see Figure 6F) for $n = 32$ parasites. P-values were calculated using Spearman Rank correlation test (with correlation coefficient ρ indicated), and lines indicate trends of the correlation found using a linear regression. The statistical significance of the correlation is shown for all data (circles) or for data that excluded two potential outliers (triangles).

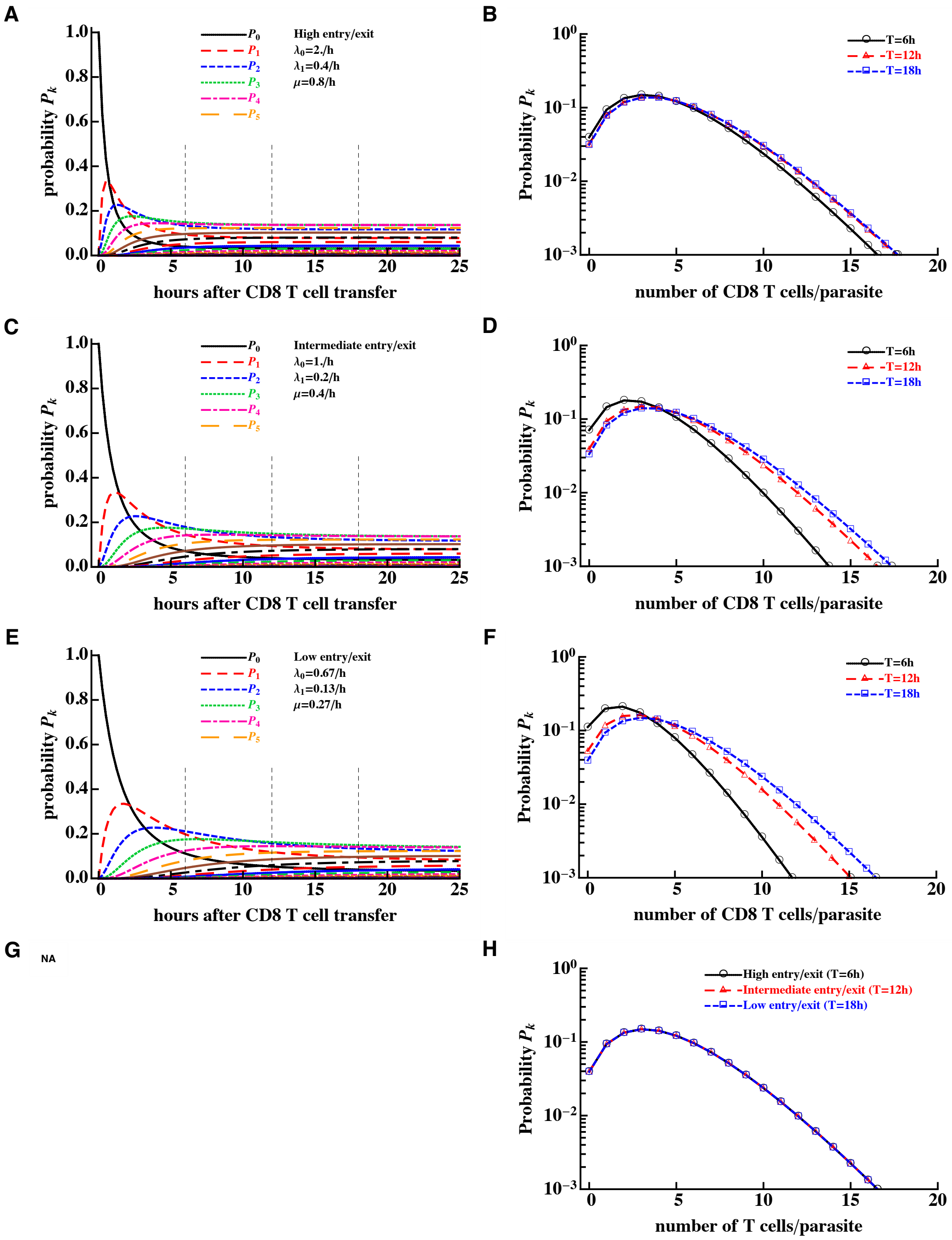

Figure S5: Scaling the entry and exit rates in the DD recruitment model and the time of measurement results in invariant prediction of the distribution of cluster sizes. We simulate cluster formation rate using the DD recruitment model (eqns. (1)–(2)) with $\lambda_k = \lambda_0 + \lambda_1 k$ and $\mu_k = \mu k$ using parameters shown in panels A, C, and E, corresponding to high, intermediate, or low rates of T cell entry into the cluster and exit from the cluster. The rates in panels C and E are scaled 2 or 3 fold from those in panel A. The distribution of cluster sizes at different times after start of clustering is shown in panels B, D, and F. Panel H shows that reducing the entry/exit rates and proportionally increasing the time when clusters are measured results in identical cluster size distribution. This suggests the invariance of the cluster size distribution to appropriately scaled rates and the time of observation.
